# Unlocking Reversible Mn^2+^/MnO_2_ Chemistry in Semisolid Slurry Electrodes for High-Performance Aqueous Zn–Mn Batteries

**DOI:** 10.1007/s40820-025-01994-9

**Published:** 2026-01-12

**Authors:** Zefang Yang, Qi Zhang, Chao Hu, Yougen Tang, Jinchi Li, Qi Wang, Wanhai Zhou, Dongliang Chao, Haiyan Wang

**Affiliations:** 1 Hunan Provincial Key Laboratory of Chemical Power Sources, College of Chemistry and Chemical Engineering, Central South University, Changsha, 410083 People’s Republic of China; 2 Laboratory of Advanced Materials, Aqueous Battery Center, State Key Laboratory of Molecular Engineering of Polymers, College of Smart Materials and Future Energy, Fudan University, Shanghai, 200433 People’s Republic of China; 3 School of Physical and Mathematical Sciences, Nanyang Technological University, Singapore, 637371 Singapore; 4 Nanotechnology Research Laboratory, Faculty of Engineering, University of Sydney, Camperdown, NSW 2006 Australia

**Keywords:** Electrolytic Zn–MnO_2_ batteries, Slurry batteries, MnO_2_ deposition/dissolution, MnO_2_ mass loading, γ-MnO_2_ phase

## Abstract

Electrolytic Zn–MnO_2_ batteries are promising candidates for safe and sustainable energy storage owing to their high voltage, environmental benignity, and cost-effectiveness. However, practical applications are hindered by the poor conductivity and the irreversible dissolution of conventional ε-MnO_2_ deposits. Herein, we report a scalable semisolid slurry electrode architecture that enables stable MnO_2_ deposition/dissolution using a three-dimensional percolating network of carbon nanotubes (CNTs) as both conductive matrix and deposition host. The slurry system promotes the formation of highly conductive γ-MnO_2_ owing to enhanced charge transfer kinetics, enabling overall dissolution rather than the localized separation typically seen in traditional electrodes. The Zn–MnO_2_ slurry cell exhibits a reversible areal capacity approaching 60 mAh cm^−2^. Moreover, the flowable nature of the slurry allows electrochemically inactive MnO_2_ formed during dissolution to be reconnected and reactivated by CNTs in the rheological network, ensuring deep utilization and cycling stability. This work establishes a slurry electrode strategy to improve electrolytic MnO_2_ reactions and offers a viable pathway toward renewable aqueous batteries for grid-scale applications.

**Supplementary Information:**

The online version contains supplementary material available at 10.1007/s40820-025-01994-9.

## Highlights

- A semisolid MnO_2_ slurry electrode enables reversible MnO_2_ deposition/dissolution within a CNT-percolated conductive network, achieving a high areal capacity of 60 mAh cm^−2^
- The slurry system promotes the formation of highly conductive γ-MnO_2_ and achieves uniform MnO_2_ dissolution through enhanced charge transfer.
- The MnO_2_ slurry electrode offers strong scalability and regenerability, retaining 100% capacity after 180 cycles and reactivating inactive MnO_2_ via percolation.

**Supplementary Information:**

The online version contains supplementary material available at 10.1007/s40820-025-01994-9.

## Graphical abstract

**Figure:**
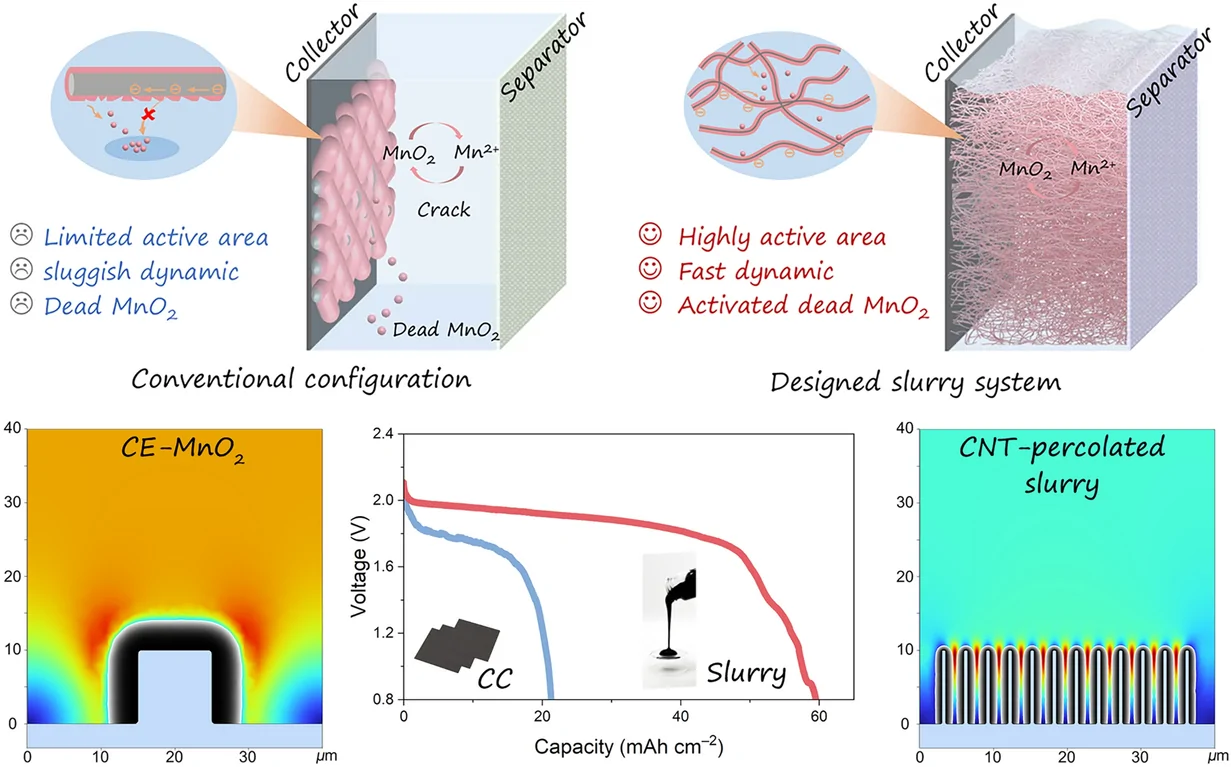

## Introduction

The objectives of low carbon and carbon neutrality are driving researchers to pursue advanced energy storage technologies, especially rechargeable batteries with the potential in grid applications [1–3]. Lithium-ion batteries (LIBs) have dominated the markets of electric vehicles and portable electronic devices but their safety and sustainability remain a concern owing to the utilization of organic electrolytes and the scarcity of lithium resources [4–8]. In recent years, aqueous batteries with nonflammable water-based electrolytes have been booming significantly for their environmental benignity, cost-effectiveness, and facile fabrication [9–11]. Of all aqueous batteries, secondary zinc–manganese (Zn–Mn) batteries have attracted widespread attention due to their high voltage and specific capacity [12–14]. The electrolytic zinc–manganese dioxide (Zn–MnO_2_) batteries operated in the acid electrolyte can deliver a high theoretical capacity of 616 mAh g^− 1^ with two-electron redox of Mn^2+^/Mn^4+^ and a high voltage (1.23 V vs. standard hydrogen electrode and 1.99 V vs. Zn/Zn^2+^) [15, 16]. However, the practical application of the electrolytic Zn–MnO_2_ batteries is hindered by sluggish charge reaction, poor electronic conductivity of MnO_2_, and phase transition, which contributes to the formation of electrochemically inactive MnO_2_ phases (dead MnO_2_) [17, 18].

Various strategies have been developed to address the above challenges, such as electrolyte additives, redox mediation, pH buffer solution, and polymorph modulation [19–24]. Notably, redox-mediated catalysis and atomic-level modulation of MnO_2_ electrolysis reactions have shown promise in improving reversibility and suppressing dead-phase accumulation [15, 16]. The reaction mechanism of electrolytic MnO_2_ is the liquid–solid transition process, as shown in the following equation [25]:

1 $${\mathrm{Mn}}^{{{2} + }} + {\text{ 2H}}_{{2}} {\text{O }} \leftrightarrow {\text{ MnO}}_{{2}} \left( {\mathrm{s}} \right) \, + {\text{ 4H}}^{ + } + {\text{ 2e}}^{ - }$$

**Fig. 1:**
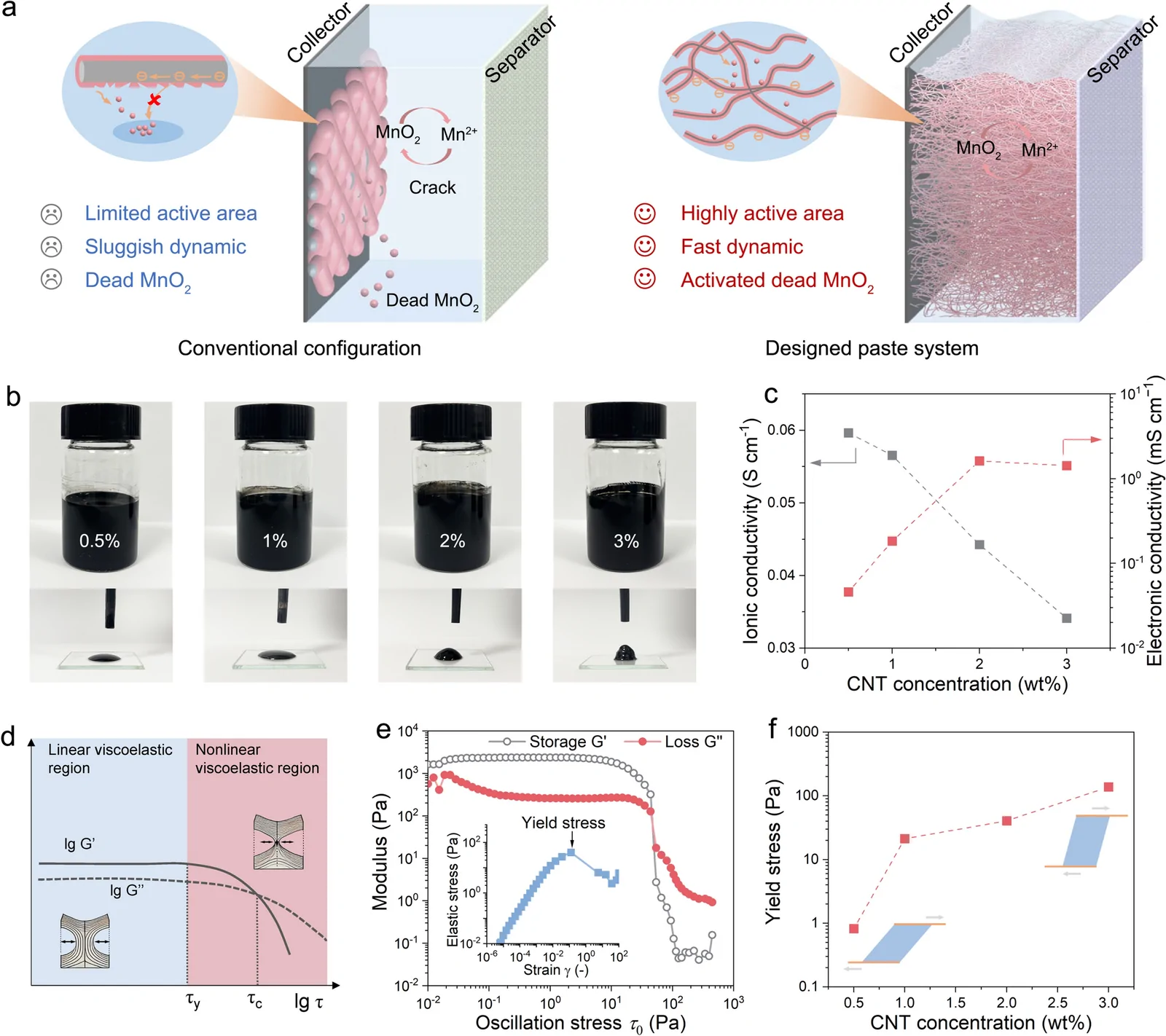
Design and preparation of MnO_2_ slurry electrodes. **a** Schematic diagram of an electrolytic Zn–MnO_2_ battery using conventional electrodes and a designed slurry electrode. **b** Digital photographs and **c** electronic and ionic conductivities of the slurry with different CNTs concentrations. **d** Typical rheological curves with different viscoelastic regions. **e** Oscillatory stress sweep measurement of slurry with a concentration of 2% CNTs. **f** Yield stress of the slurry with different CNTs concentrations

This reaction is highly dependent on the reaction interface, where a high specific surface area substrate promotes a high MnO_2_ deposition capacity [26]. Carbon cloth (CC) and carbon felt are currently the most commonly used host materials for MnO_2_ deposition [27]. However, in conventional electrolytic Zn–MnO_2_ (CE-MnO_2_) cells (Fig. 1a), MnO_2_ is preferentially dissolved on the highly active regions of these carbon substrates during discharge due to the poor electrical conductivity of MnO_2_ and the limited surface area of the substrate, resulting in unavoidable active MnO_2_ falling out into the electrolyte [28, 29]. It is noteworthy that the high conductivity and specific surface area of the substrate with abundant three-dimensional (3D) channels are undeniable for stable MnO_2_ deposition/dissolution, which would induce a thin MnO_2_ deposition layer to shorten the electron transport distance and achieve the overall dissolution of MnO_2_ [23]. Li et al. reported that carbon nanotubes (CNTs)-modified carbon felt (CNT–CF) substrates prepared by a high-temperature vapor-phase growth strategy can effectively improve MnO_2_ deposition capacity due to the high specific surface area of CNTs [26]. However, the complexity and high cost of CNT–CF are not conducive to scale-up industrial fabrication. Can commercialized CNTs improve MnO_2_ deposition/dissolution? During MnO_2_ dissolution, a portion of the MnO_2_ migrates into the electrolyte to form dead MnO_2_ species that physically disrupt the conductive pathways. When adequate CNTs are uniformly dispersed within the electrolyte to form a conductive slurry electrode and concurrently function as a deposition substrate for MnO_2_, the electrochemically inert MnO_2_ can be reactivated through electron transfer from adjacent CNTs during the discharge process. The development of electrolytic Zn–MnO_2_ slurry batteries shows great potential in scalable energy storage, but their implementation remains unreported.

In this paper, we construct for the first time a semisolid MnO_2_ slurry cell that employs uniformly dispersed commercial CNTs into the electrolyte to establish a percolating electron-conductive network throughout the catholyte. The CNTs act simultaneously as the electron conducting scaffold and the dynamic host for MnO_2_ deposition, which overcomes the reactive area limitations of traditional carbon-based substrates (carbon felt and CC) to promote high-loading MnO_2_ deposition and dissolution. By combining X-ray computed tomography (X-CT), in situ Raman spectroscopy, in situ optical visualization, and theoretical calculations, it is revealed that MnO_2_ deposition within the slurry shows a highly conductive γ-MnO_2_ phase and undergoes uniform dissolution, in contrast to the localized dissolution behavior of common ε-MnO_2_ with poor conductivity in CE-MnO_2_ batteries. As a result, the Zn–MnO_2_ slurry cell delivers a reversible areal capacity approaching 60 mAh cm^− 2^. Furthermore, electrochemically inactive MnO_2_ particles generated during the dissolution process are reactivated through electron transfer from neighboring CNTs within the rheological network. A reversible capacity of 21.3 mAh cm^− 2^ is recovered for the post-cycled Zn–MnO_2_ slurry cell, which reflects the inherent capability of the slurry electrode design to reactivate electrochemical inactive MnO_2_. Building upon previous approaches that utilized redox mediation and catalytic kinetics to stabilize MnO_2_ dissolution, we here establish a scalable slurry-based framework that offers broader implications for the design of high-performance aqueous batteries.

## Experimental Section

### Materials

The chemicals and materials in this work are all commercially available and used without purification. Manganese sulfate monohydrate (MnSO_4_·H_2_O), zinc sulfate heptahydrate (ZnSO_4_·7H_2_O), sulfuric acid (H_2_SO_4_), and polyvinylpyrrolidone (PVP) were purchased from Shanghai Aladdin Biochemical Technology Co., LTD. Carbon cloth, carbon nanotubes (CNTs), anion exchange membrane (AEM), stainless mesh, graphite plate, copper foil, and zinc foil were obtained from Taobao.com.

### Preparation of Electrolytic MnO_2_ Slurry

PVP with a content of 1 wt% was dissolved into 1 M MnSO_4_ and 0.1 M H_2_SO_4_ solution. After that, different concentrations of CNTs were added to the above solution by ultrasonic dispersion to obtain a uniform conductive slurry.

### Characterizations

The structure and morphology of electrolytic MnO_2_ were characterized by X-ray diffraction (XRD, PANalytical/2Empyrean 2), scanning electron microscope (SEM, JSM-7610FPlus), and transmission electron microscope (TEM, JEOL JEM-F200). X-ray photoelectron spectroscopy (XPS, ESCALAB250Xi) was used to analyze the manganese oxidation state. MnO_2_ deposition/dissolution evolution was recorded by in situ Raman spectroscopy (Renishaw/inVia Reflex) and in situ optical observations (Nikon Ti2-A). The slurry dispersity before and after MnO_2_ deposition was analyzed using the X-ray microscopic computed tomography technique (Xradia 620 Versa). The rheological performance of the slurry with different concentrations of CNTs was measured on a stress-controlled rheometer (Haake Mars60). The oscillatory stress region of elastic deformation was measured based on the method proposed by Walls et al. [30]. The Mn^2+^ ion concentration during MnO_2_ deposition and dissolution was recorded by the electrochemical digital holography (EDH) using Mach–Zehnder interferometer optical setup. The elastic contribution to the total oscillatory stress was calculated by multiplying the elastic modulus by the oscillating stress amplitude.

### Electrochemical Measurements

The Zn–MnO_2_ slurry cell was assembled in a custom-made cell (Fig. S5) with hydrophobic CC as the current collector, zinc foil as the anode, 1 M ZnSO_4_ as anolyte, and glass fiber as the anodic separator. The AEM was placed between the slurry and the anolyte. For the assembly of CE-MnO_2_ cell with AEM, two pieces of glass fibers separated by AEM were acted as cathodic and anodic separators with 1 M MnSO_4_ and 0.1 M H_2_SO_4_ as catholyte, 1 M ZnSO_4_ as anolyte, hydrophilic CC as substrate, and zinc foil as the anode. The assembly of the CE-MnO_2_ cell without AEM was the same as for CE-MnO_2_ cell with AEM, except that 1 M MnSO_4_, 0.1 M H_2_SO_4_, and 1 M ZnSO_4_ solution was used as electrolyte. For the Zn–MnO_2_ slurry flow cell, a piece of AEM with an area of ~ 2 × 2 cm^2^ was used to separate anolyte and slurry. The active area was based on the carbon substrate (1 × 1 cm^2^), and the thickness of the slurry chamber was set to 2 mm. In the electrochemical tests, 15 mL slurry was circulated between the cathode chamber and the storage tanks with a peristaltic pump at a flow rate of 5 mL min^− 1^. The CNTs film electrode was prepared by mixing 80 wt% CNTs, 10 wt% Ketjen black, and 10 wt% polytetrafluoroethylene (PTFE) binder in an agate mortar with ethanol as the dispersing solvent. After sufficient grinding, the mixture gradually transformed into a plasticine-like material, which was subsequently rolled onto a titanium mesh and dried at 60 °C for 6 h. Linear sweep voltammetry (LSV) measurements were conducted with hydrophilic CC as the work electrode, platinum mesh as the counter electrode, and Ag/AgCl as the reference electrode at a scan rate of 5 mV s^− 1^. Cyclic voltammetry (CV) profiles were recorded in three-electrode configurations with hydrophilic CC as the working electrode, platinum mesh as the counter electrode, and Ag/AgCl as the reference electrode in 1 M MnSO_4_, 0.1 M H_2_SO_4_, and 1 M ZnSO_4_ electrolyte. The electrochemical impedance spectroscopy (EIS) was tested with an amplitude of 5 mV in a frequency range from 0.01 Hz to 0.1 MHz. LSV, CV, and EIS were carried out on a CHI 760D electrochemical workstation. The constant voltage charge and galvanostatic discharge tests were performed on a Neware battery testing system.

### Theoretical Calculations

Density functional theory (DFT) calculations were carried out in the Vienna ab initio simulation package (VASP) with the generalized gradient approximation proposed by Perdew, Burke, and Ernzerhof (GGA-PBE) as the exchange correlation function and the projection augmented wave (PAW) potentials [31–33]. The surfaces of ε-MnO_2_ and γ-MnO_2_ phases are composed of 3 O–Mn–O layers. In the geometrical optimization, the structural convergence was set to 10^− 5^ eV for the energy, 0.01 eV Å^− 1^ for the maximum force, and 450 eV for the cutoff energy. A vacuum layer with 18 Å thick in the *z*-direction was constructed to circumvent the interactions of the periodic surfaces [34]. The free energies of hydrogen adsorption and water desorption on the MnO_2_ surfaces are calculated by the following formula:

2 $$\Delta G = \Delta E_{{{\mathrm{DFT}}}} + \Delta E_{{{\mathrm{ZPE}}}} {-}T\Delta S$$

where *ΔE*_DFT_, *ΔE*_ZPE_, *T* and *ΔS* are the DFT electron energy difference at each step, the zero-point energy correction, temperature, and entropy change, respectively [35]. The binding energy of Mn and H_2_O on CNTs or CC was calculated by the following Eq. (3):

3 $$E = E_{{{\mathrm{total}}}} {-}E_{{{\mathrm{slab}}}} {-}E_{{{\mathrm{ad}}}}$$

where *E*_total_, *E*_slab_ and *E*_ad_ are the total energy of Mn or H_2_O adsorbed on these carbon surfaces, the energy of CNTs or CC surface, and the energy of adsorption of Mn or H_2_O, respectively. The interface energy (*γ*_ab_) between materials *a* (ε-MnO_2_ and γ-MnO_2_) and *b* (CNTs and CC) is defined as the energy difference between an interface system and the bulk energy of these materials.

4 $$\gamma_{ab} = \frac{{E_{ab} - n_{a} E_{a} - n_{b} E_{b} }}{A}$$

where *E*_ab_, *E*_a_ and *E*_b_ are the total energy of the interface, the energy of bulk *a* and bulk *b*, respectively. The *n*_i_ (*i* = *a*, *b*) denotes the ratio of bulk atoms to interfacial atoms. The *A* refers to interfacial area.

### Simulations

MnO_2_ deposition/dissolution evolution on CC and CNTs was simulated by the finite element method in Comsol Multichsics 6.0 [36]. The tertiary current distribution coupled with the lever set deformation in a two-dimensional transient model was applied. A square with a size of 10 × 10 μm was constructed at the cathode interface to act as a CC fiber. For CNTs, an array consisting of rods with a size of 0.5 × 10 μm at intervals of 2.5 μm was built. MnO_2_ deposition condition was set to a current density of 1 mA cm^− 2^ for 3 h. The key parameters used in the simulations are listed in Table S1. Electrode kinetics were decided by the Butler–Volmer equation, which determines the local interfacial current density.

5 $$i_{loc} = i_{0} \left( {\exp \left( {\frac{{{\upalpha }_{a} F{\upeta }}}{RT}} \right) - \frac{{c_{{Mn^{2 + } }} }}{{c_{{Mn^{2 + } ,ref}} }}\exp \left( { - \frac{{{\upalpha }_{c} F{\upeta }}}{RT}} \right)} \right)$$

where *i*_0_, *α*_a_, *α*_c_, *F*, *η*, and ${\mathrm{C}}_{{Mn}^{2+}}$ are the exchange current density, the anode transfer coefficient, the cathode transfer coefficient, Faraday’s constant, the overpotential, and the Mn^2+^ concentration, respectively. The overpotential is defined as follows:

6 $$ \eta = \varphi _{s} - \varphi _{l} - {\mathrm{E}}_{eq}$$

where *φ*^s^, *φ*^1^, and *E*_eq_ are the solid and electrolyte potentials, and the equilibrium potential, respectively. The level set interface is used to track the deformation of the cathode surface during the deposition process. The level set interface automatically establishes equations governing the movement of the interface between the liquid electrolyte and the solid electrode. The transport of level set variables is described by Eq. (7):

7 $$\frac{\partial \phi }{{\partial t}} + u \cdot \nabla \phi = {\upgamma }\nabla \cdot \left( {{\upvarepsilon }\nabla \phi - \phi \left( {1 - \phi } \right)\frac{\nabla \phi }{{\left| {\nabla \phi } \right|}}} \right)$$

where *ε* controls the thickness of the interface (*ε* = *h*_max_/4, with hmax the maximum mesh size) and *γ* is a reinitialization parameter related to the maximum velocity. The delta function in the level set method is approximated as follows:

8 $${\updelta } = 6\left| {\phi \left( {1 - \phi } \right)} \right|\left| {\nabla \phi } \right|$$

The velocity field governing the motion of the deposition front is linked to the local current density.

9 $$u = n \cdot \left( { - \frac{{i_{loc} M_{{MnO_{2} }} }}{{2F{\uprho }_{{MnO_{2} }} }}} \right)$$

where ${M}_{Mn{O}_{2}}$ and ${\uprho }_{Mn{O}_{2}}$ are the molar mass and density of MnO_2_, respectively. The interface normal vector is as follows:

10 $$n = \frac{\nabla \phi }{{\left| {\nabla \phi } \right|}}$$

### Yield Stress Calculations

Sedimentation in a yield stress fluid occurs in a small region around the particles where either gravity or yield stress is present [37]. The dimensionless yield stress is defined as the ratio of the fluid yield stress to the stress exerted by the particles on the fluid (e.g., particle gravity) when simulating the yield zone of the particles during flow cessation and fluid solidification [38, 39].

11 $$Y_{cr} = \frac{{2\pi R^{2} \sigma_{y} }}{{\frac{4}{3}\pi R^{3} \left( {\rho_{p} - \rho_{l} } \right)g}} = \frac{{1.5\sigma_{y} }}{{R\left( {\rho_{p} - \rho_{l} } \right)g}}$$

where *g*, *ρ*_p_, *ρ*_l_, and *R* are the gravitational acceleration, MnO_2_ particle density, fluid density, and particle radius, respectively. *R* is considered to be the radius of the MnO_2_ particles, approximately 1 μm from TEM images. MnO_2_ has a density of 5000 kg m^− 3^. The fluid density is taken to be 2000 kg m^− 3^.

## Results and Discussion

### Preparation of Characterization of Slurry Electrodes

The electrochemical stability windows (ESW) of the electrolytes for deposition and dissolution of MnO_2_ and zinc were investigated by linear sweep voltammetry measurement. One mole per liter (M) of MnSO_4_ with 0.1 M H_2_SO_4_ additive is used as the catholyte for the electrolytic MnO_2_ since MnO_2_ deposition/dissolution reversibility can be significantly improved with the addition of a small amount of H_2_SO_4_ [25]. As shown in Fig. S1a, the MnO_2_ deposition onset voltage in the catholyte is 1.1 V (vs. Ag/AgCl), much lower than that of O_2_ evolution (1.85 V). The anolyte with 1 M ZnSO_4_ presents H_2_ evolution and zinc deposition potential over − 1.07 and − 0.94 V, respectively. The ESW is about 2.92 V, and the minimum electrolysis voltage is 2.14 V for the electrolytic Zn–MnO_2_ battery. The hybrid Zn–MnO_2_ cell delivers a practical voltage of 1.94 V (Fig. S1b), which is conducive to achieving Zn-based energy storage batteries with high energy density.

CNTs with different contents are added to the catholytes to form uniform slurry electrodes (Fig. 1b). The CNTs network can act as both conductive pathways for electron transport and MnO_2_ deposition hosts. The electronic conductivity of the slurry gradually increases with the addition of CNTs and tends to be stable at the CNTs content of 2% (Figs. 1c and S2). Conversely, the slurry ionic conductivity decreases from 0.059 to 0.034 S cm^− 1^ when the concentration of CNTs is increased from 0.5% to 3% (Fig. S3), which is ascribed to the increased slurry viscosity upon the addition of CNTs. The appropriate viscosity of the slurry is essential to counteract the gravity of the conductive agents and the MnO_2_ deposits in the slurry from sedimentation during rest and reaction. Quantifying the transition of the slurry from viscoelastic behavior to sedimentation through rheological curves provides a basis for understanding the critical stress at which structural breakdown occurs (Fig. 1d). We further measure the static yield stress of the slurry by using oscillatory shear flow deformations to provide insight into the stress-bearing CNTs network (Figs. 1e and S4). The yield stress of the slurry is sensitive to increasing CNTs concentration, and it increases with the addition of more CNTs (Fig. 1f). Calculations for the critical dimensionless yield stress of the slurry are conducted (the detailed calculation process is shown in the Supporting Information) [39].

For the slurry with CNTs concentrations ranging from 0.5% to 3%, the *Y*_cr_ value is decided to be ~ 40 to ~ 7000, several orders of magnitude larger than the critical *Y*_cr_ value for the onset of sedimentation (0.05 ≤ *Y*_cr_ ≤ 0.14), suggesting the potential prevention of the formed MnO_2_ particles sedimentation in the slurry [38]. The electrochemical charge and discharge tests of slurry were carried out on a homemade battery device (Fig. S5). As shown in Fig. S6, the slurry cathode with 2% CNTs can deliver a maximum capacity of 1.8 mAh cm^− 2^ with a discharge platform of 1.9 V in comparison with those of 0.5% (1.66 mAh cm^− 2^), 1% (1.68 mAh cm^− 2^), and 3% CNTs (1.72 mAh cm^− 2^). The high content of CNTs in the slurry not only builds a tight conduction network for electron transport but also avoids the sedimentation of the slurry through the resulting high-yield stress. However, the ion transport of the slurry is greatly hindered by the high viscosity resulting from the excessive addition of CNTs. Based on the above analysis, the optimized slurry with a concentration of 2% CNTs is further studied unless otherwise specified. Besides, the conductive substrate for the slurry electrode is also optimized by the MnO_2_ deposition potential (Figs. S7 and S8).

**Fig. 2:**
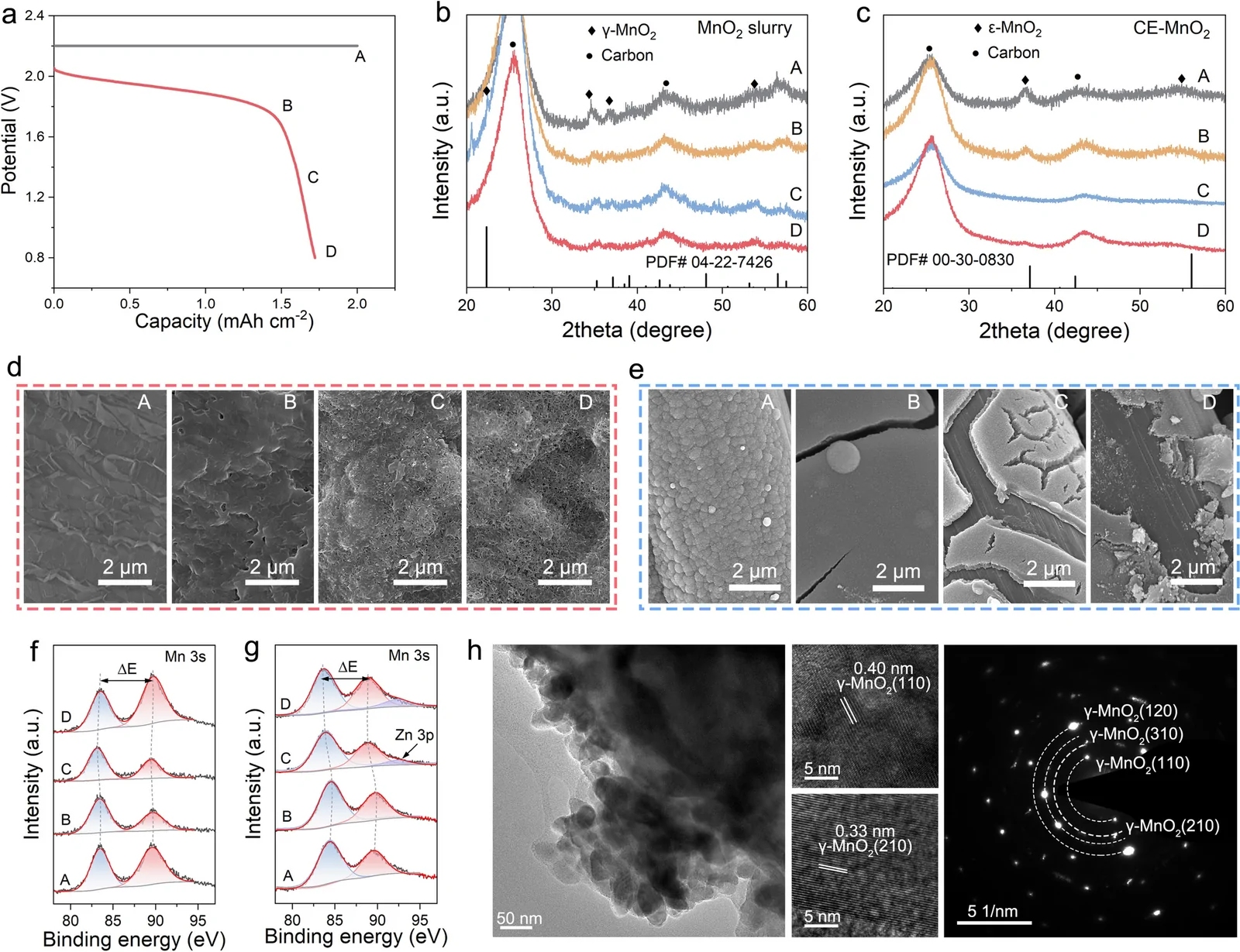
Characterizations of MnO_2_ deposition/dissolution in the slurry. **a** Voltage curves of MnO_2_ electrode at constant voltage charge and galvanostatic discharge, A, B, C and D represent charged for 2 mAh cm^− 2^, discharge to 1.7, 1.4 and 0.8 V, respectively. **b, c** XRD patterns, **d, e** SEM images and **f, g** Mn 3*s* spectra of **b, d, f** MnO_2_ slurry electrode and **c, e, g** CE-MnO_2_ cell without AEM at different charge and discharge states. **h** TEM images, high-resolution TEM images and the selected area electron diffraction patterns of MnO_2_ deposition in MnO_2_ slurry cells

The structure, morphology, and chemical compositions of the deposited MnO_2_ in the slurry cathode were investigated by X-ray diffraction (XRD), scanning electron microscopy (SEM), and X-ray photoelectron spectroscopy (XPS) at different charge/discharge states (Fig. 2a). The charged MnO_2_ in the slurry cathode is confirmed to be of γ-MnO_2_ (JCPDS card 04–022–7426) phase (Fig. 2b), which differs from the previously reported ε-MnO_2_ (JCPDS card 00–030–0820) in CE-MnO_2_ batteries [15, 40–43]. No obvious phase changes are observed in the subsequent discharge to 1.7, 1.4, and 0.8 V, but the peak intensity in XRD patterns gradually diminishes with the discharge. However, MnO_2_ deposition in CE-MnO_2_ cells exhibits a typical ε-MnO_2_ and higher crystallinity in the cell using AEM than that without the AEM (Figs. 2c and S9). MnO_2_ particles with a size of approximately 1 μm are observed in SEM images of the MnO_2_ slurry cell after charge, and they tend to dissolve entirely during the subsequent discharge process (Figs. 2d and S10). However, both CE-MnO_2_ cells with and without AEM exhibit nanosphere morphology on CC after MnO_2_ deposition (Figs. 2e, S11, and S12). Localized dissolution is found in CE-MnO_2_ cells without AEM due to the selected dissolution of highly active regions during discharge, leading to the formation of dead MnO_2_. Besides, the low electronic conductivity of ε-MnO_2_ is also responsible for incomplete MnO_2_ dissolution in CE-MnO_2_ cells. The electronic conductivity of γ-MnO_2_ is two orders of magnitude higher than that of ε-MnO_2_, which is conducive to the reversibility of the MnO_2_ cathode [23].

The spin-splitting energy (∆E) is calculated to be about 5.2 eV in the Mn 3s XPS spectra of CE-MnO_2_ cells with and without AEM at different states by the single peak fitting and even exceeds 6 eV for MnO_2_ slurry cell, which suggests the presence of multiple splitting in these Mn 3s spectra (Figs. 2f, g, and S13) [44]. After discharge to 1.4 V, a Zn 3p appears in the Mn 3s spectra of CE-MnO_2_ cell without AEM due to the insertion of Zn^2+^ with free shuttle in electrolytes, in accordance with the previously reported works [16, 20]. The ∆E values of the deconvoluted Mn 3s spectra in the CE-MnO_2_ cell with and without AEM are about 4.7/5.5 eV and 4.7/5.8 eV, respectively, corresponding to MnO_2_ and Mn^3+^ intermediates (Fig. S14) [16, 45]. However, the Mn 3*s* spectra of the MnO_2_ slurry cells can be deconvoluted to give ∆E values of about 4.7/6.7 eV, which is attributed to the formation of MnO_2_ and the residues of the electrolyte [44, 45]. The Mn–O–Mn and Mn–OH bonds in the O 1*s* of the CE-MnO_2_ cell with AEM are shifted to high binding energies and show significant fluctuations during MnO_2_ dissolution compared to those of the one without AEM, which is due to the incorporation of H^+^/Zn^2+^ ions in the discharge reactions (Fig. S15) [46, 47]. Furthermore, MnO_2_ slurry cells present higher binding energy shifts for Mn–O–Mn and Mn–OH bonds compared to CE-MnO_2_ cells with and without AEM, but remain slightly changed during discharge, probably due to the high Mn oxidation state in the MnO_2_ slurry.

Transmission electron microscopy (TEM) was used to visualize the microscopic structure of MnO_2_ deposition in those electrolytic Zn–MnO_2_ cells. As shown in Fig. 2h, ellipsoid-shaped MnO_2_ nanoparticles with high crystallinity are presented in MnO_2_ slurry cells after the first charge and indexed as γ-MnO_2_ phase. However, the CE-MnO_2_ cell without AEM exhibits irregular crystalline MnO_2_ nanoparticles with a large number of amorphous structures (Fig. S16), which is attributed to the interference of the unstable pH on the MnO_2_ deposition reaction induced by H_2_ evolution at the zinc anode [48]. In contrast, the well-crystallized MnO_2_ nanoparticles are observed in CE-MnO_2_ cells with AEM. The typical interplanar spacing and diffraction patterns of MnO_2_ deposits in CE-MnO_2_ cells with and without AEM can be identified as ε-MnO_2_, which is consistent with the XRD results. An undisturbed proton concentration can facilitate the reaction Eq. (1) in a positive direction to gain crystallographic MnO_2_ as multiple disproportionation reactions occur with an increase in pH during the MnO_2_ deposition [49]. Additionally, the formation of dense MnO_2_ deposits with high crystallinity in slurry cells is benefited by a highly conductive γ-MnO_2_ phase and a 3D conducting CNTs network, which enables the overall MnO_2_ dissolution. Also, the highly specific surface area of CNTs is favorable for the formation of a thin MnO_2_ layer to realize a stable MnO_2_ dissolution (Fig. S17).

### Electrochemical performance of the Slurry Electrode

**Fig. 3:**
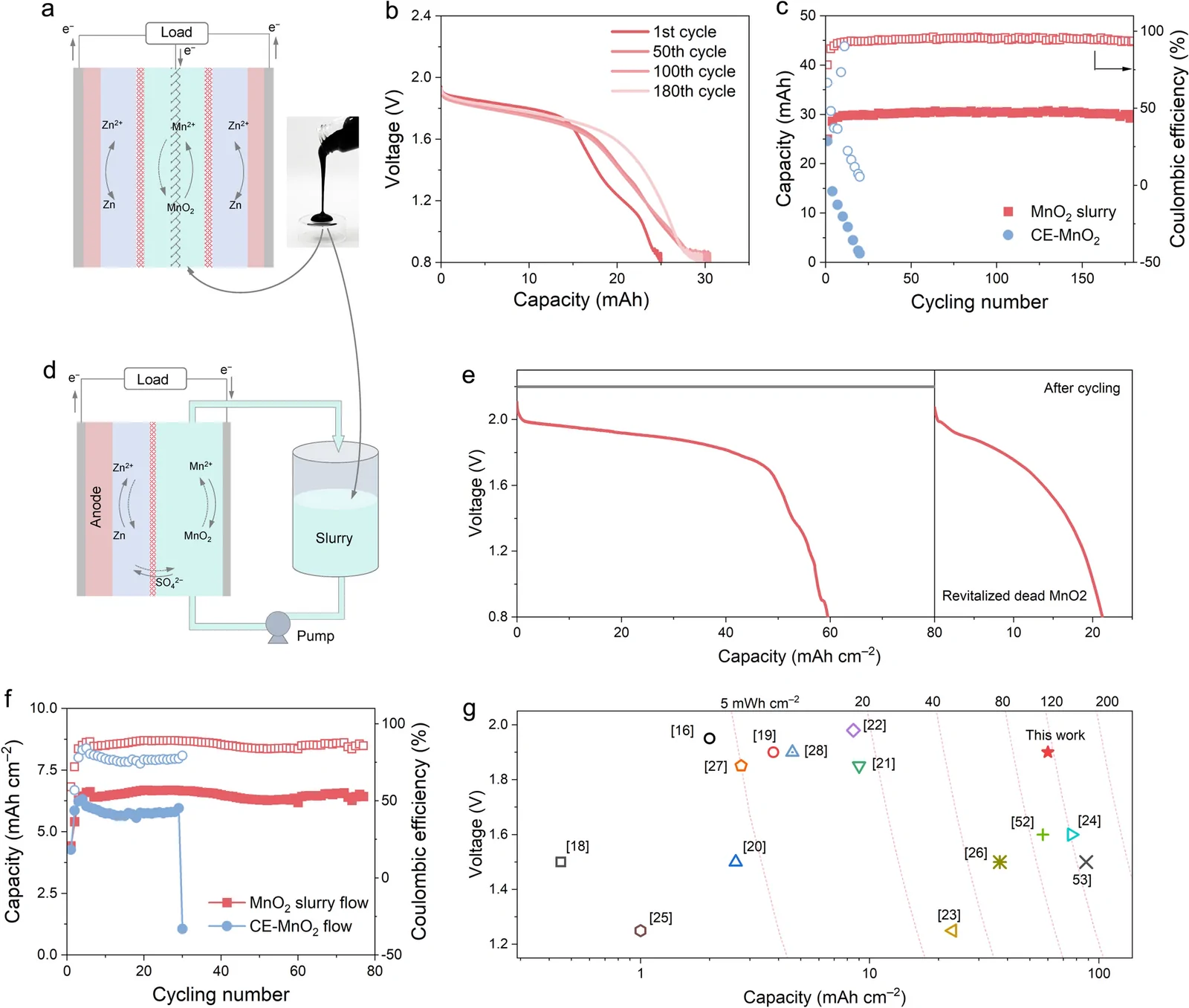
Scale-up of the Zn–MnO_2_ slurry cell. **a** Schematic diagram of the static Zn–MnO_2_ slurry battery. The inset shows a digital photograph of flow slurry. **b** Discharge curves of the enlarged Zn–MnO_2_ slurry cell at 128 mA for 32 mAh. **c** Cycling stability of the scale-up CE-MnO_2_ and Zn–MnO_2_ slurry cell. **d** Schematic diagram of the Zn–MnO_2_ slurry flow battery. **e** The first charge and discharge (left side) curves and post-cycled (right side) discharge profiles of the Zn–MnO_2_ slurry flow battery at a discharge current density of 1.5 mA cm^− 2^; the cycling condition is set to 1.5 mA cm^− 2^ and 15 mAh cm^− 2^ (Fig. S24). **f** Cycling performance of Zn–MnO_2_ slurry and CE-MnO_2_ flow batteries at 1.5 mA cm^− 2^ and 7.5 mAh cm^− 2^. **g** Comparison of discharge capacity and voltage of Zn–MnO_2_ slurry cells with previously reported electrolytic MnO_2_-based cells

Considering the proposed electrolytic MnO_2_ slurry system, we first constructed the static Zn–MnO_2_ slurry cell in a custom-made Perspex cell and performed the electrochemical measurements (Fig. 3a). To suppress the O_2_ evolution reaction on the cathode, a chronoamperometric (constant potential) charge with a potential of 2.2 V was applied to the Perspex cell, along with a galvanostatic discharge process. The MnO_2_ slurry cell can operate steadily for 600 cycles with an average Coulombic efficiency (CE) of 93.48% at a charge capacity of 0.5 mAh cm^− 2^ and a discharge current density of 1 mA cm^− 2^ (Fig. S18a). However, the CE-MnO_2_ cell without AEM shows a severe capacity decay after 109 cycles and an average CE of 79.73%, which is due to the poor electronic conductivity of ε-MnO_2_, H_2_ evolution and the interference of Zn^2+^ ions [23, 50]. With the introduction of AEM, the cycle life of the CE-MnO_2_ cell is extended to 197 cycles with an average CE of 94.66%, but the CE fluctuates sharply between 110 and 197 cycles. When the discharge current density is increased from 1 to 10 mA cm^− 2^, both CE-MnO_2_ cells with and without AEM fail immediately at 10 mA cm^− 2^ because of the sluggish kinetic reactions (Fig. S18b). In contrast, the rate capability of the MnO_2_ slurry cell is superior to that of CE-MnO_2_ cells and a capacity retention of 80% is maintained at 10 mA cm^− 2^ (vs. 1 mA cm^− 2^) in the MnO_2_ slurry cell. The MnO_2_ slurry cells show great prospects in low-cost recycling and regeneration owing to the freestanding fluidity slurry and facile liquid injection process (Fig. S18c-e). The discharge behavior of commercial MnO_2_ powder in slurry further suggests the ability of the slurry electrode to reactivate inactive MnO_2_ (Fig. S19). To evaluate the potential of electrolytic MnO_2_ slurry cells for practical applications, we develop reactive slurry electrodes with a size of 4 × 4 cm^2^ to scale up the energy storage capacity. The fabrication of practical Zn–MnO_2_ slurry cells is easily realized by injecting the slurry into Perspex cells due to the flowable slurry without a binder (Fig. S20). For the preparation of zinc anodes (ZnSn@Cu), zinc with a capacity of 3 mAh cm^− 2^ is pre-plated on chemically tinned copper (Sn@Cu) foil (Fig. S21) [51]. The assembled practical Perspex slurry (ZnSn@Cu-MnO_2_) cell with MnO_2_ slurry cathode and ZnSn@Cu anode shows a plateau voltage of 1.8 V and capacity of 25 mAh after the first charging at 2.2 V for 32 mAh (Fig. 3b). After activation for 10 cycles, the ZnSn@Cu-MnO_2_ cell delivers a capacity of 30 mAh and maintains a capacity retention of 100% over 180 cycles while the CE-MnO_2_ cell with ZnSn@Cu anode fails sharply after 20 cycles (Figs. 3c and S22). Multilayer slurry electrodes with a simplified assembly process were designed to achieve high-capacity and high-voltage slurry cells. The ZnSn@Cu-MnO_2_ cell with three slurry electrodes in parallel can operate steadily for 180 cycles and retain a total capacity of 86 mAh (Fig. S23). The electrochemical property of the MnO_2_ slurry was further evaluated in a flow cell configuration (Figs. 3d and S24). After the charging for a capacity of 80 mAh cm^− 2^, MnO_2_ deposited on CNTs is uniformly redistributed in the slurry reservoir via peristaltic pumping. Upon discharge, the Zn–MnO_2_ slurry flow cell exhibits a reversible capacity approaching 60 mAh cm^− 2^ (Fig. 3e), while only a capacity of 21 mAh cm^− 2^ is recovered for the CE-MnO_2_ flow cell (Fig. S25). Continuous slurry agitation can enhance the electronic percolation between MnO_2_ and CNTs, improving overall electric contact. For the post-cycling slurry cell, the peristaltic pump maintains stable operation for 1 h in the absence of electrochemical cycling, after which 21.3 mAh cm^− 2^ of inactive MnO_2_ is effectively reactivated (Fig. S26). As shown in Fig. 3f, the Zn–MnO_2_ slurry flow cell sustains a capacity of 6.43 mAh cm^− 2^ over 77 cycles with a CE of 85.7%, in contrast to the CE-MnO_2_ cell, which fails after 29 cycles with a residual capacity of 5.7 mAh cm^− 2^ (a CE of 80%). The CE-MnO_2_ system achieves an areal energy density of 114 mWh cm^− 2^, outperforming previously reported Mn^2+^/MnO_2_-based configurations (Fig. 3g) [52, 53]. The slurry cells are connected in series to elaborate their practicability. It is demonstrated successfully to charge mobile phones and drive mini electric fans (Fig. S27). The designed electrolytic Zn–MnO_2_ slurry battery shows great competitiveness in the development of high-safety, high-voltage aqueous zinc-based batteries for grid energy storage applications.

### MnO_2_ Deposition and Dissolution Behavior in the Slurry Electrode

The post-cycled cells were investigated to determine the reaction characteristics of the MnO_2_ slurry cells. The discharge plateau voltage of the MnO_2_ slurry cells at half discharge capacity shows a reduction of 14.5 mV from the 5th to the 20th cycle (Fig. S28). For CE-MnO_2_ cells with and without AEM, the plateau voltage is declined by 24.1 and 60 mV, respectively. The severe voltage drop in CE-MnO_2_ cell without AEM can be described as the H_2_ evolution-induced insufficient MnO_2_ dissolution reaction with low proton concentration (Fig. S29). This phenomenon is further confirmed by finite element analysis (FEA) simulations of the electrolytic MnO_2_ discharge process considering the involvement of the H_2_ evolution reaction at the anode (Fig. S30). SEM images reveal that MnO_2_ tends to dissolve partially in the post-cycled CE-MnO_2_ cells with and without AEM (Figs. S31 and S32). Large amounts of dead MnO_2_ are exposed in these post-cycled CE-MnO_2_ cells due to the thick MnO_2_ layer with low electronic conductivity. However, after cycling, the MnO_2_ slurry cells remain uniform deposition with micron-sized particles and highly reversible dissolution behavior (Fig. S33), as evidenced by the exposure of CNTs during discharge. No significant structural changes are observed in the XPS spectra of the MnO_2_ slurry cells after cycling, while the CE-MnO_2_ cells with and without AEM display Zn^2+^ insertion and additional peaks in the O 1*s* spectra, respectively (Fig. S34).

**Fig. 4:**
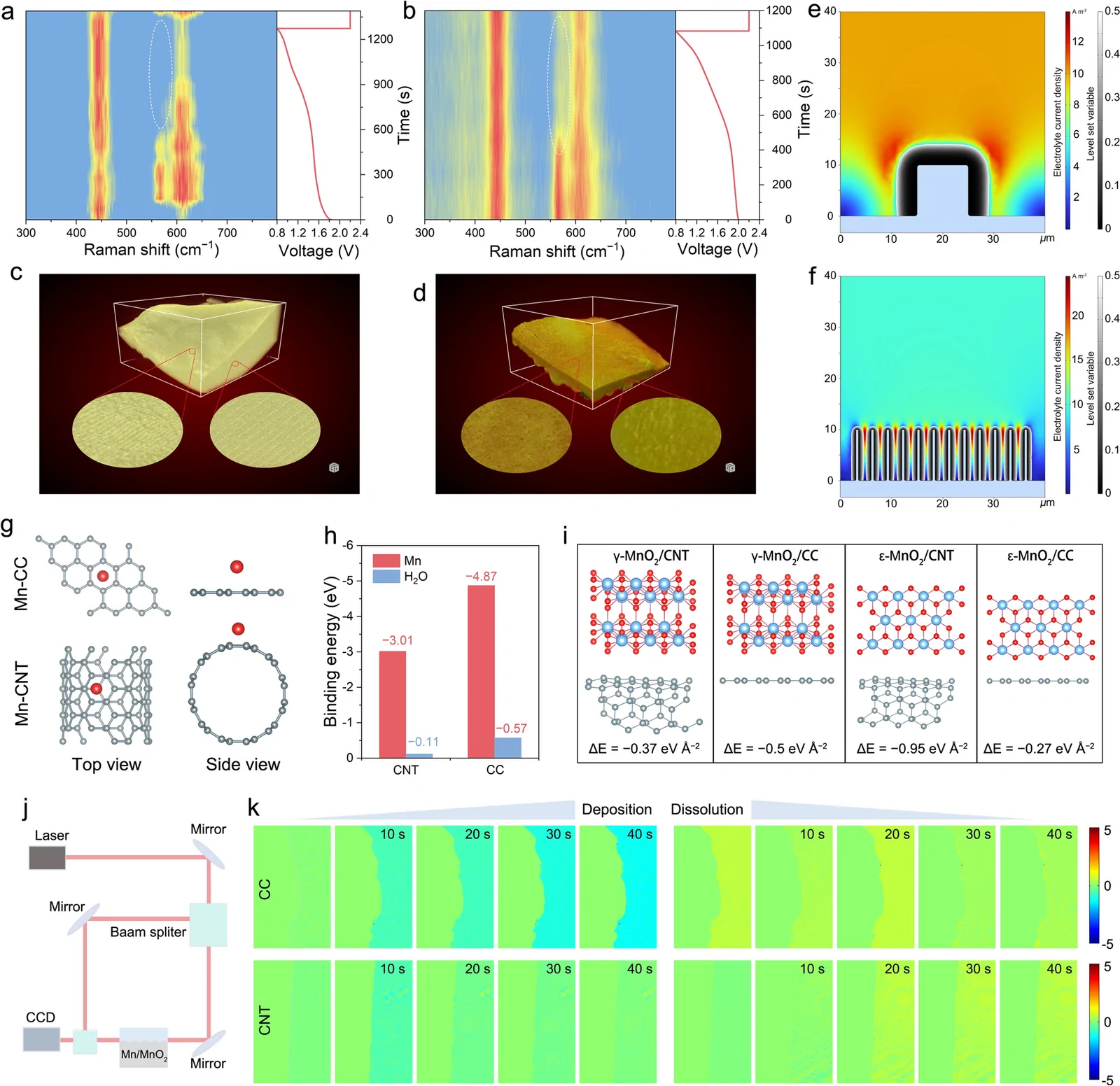
Reaction mechanism of MnO_2_ slurry cells. In situ Raman spectra of **a** Zn–MnO_2_ slurry and **b** CE-MnO_2_ cells without AEM during discharge and charge. X-CT images of the slurry electrode **c** before and **d** after MnO_2_ deposition. FEA simulations of MnO_2_ deposition on **e** CC and **f** CNTs. **g** Structural models and **h** the calculated results of Mn atoms adsorbed on CC and CNTs.** i** Interfacial structure and energy of γ-MnO_2_ on the CNTs (γ-MnO_2_/CNT) and CC (γ-MnO_2_/CC), and ε-MnO_2_ on the CNTs (ε-MnO_2_/CNTs) and CC (ε-MnO_2_/CC). **j** Schematic illustration of in situ EDH for electrochemical measurements. **k** In situ EDH of MnO_2_ deposition and dissolution on CC and CNTs

In situ Raman spectroscopy was recorded to investigate the structural evolution of MnO_2_ dissolution/deposition in both MnO_2_ slurry and CE-MnO_2_ cells within a homemade configuration (Fig. S35). The Mn–O vibrational peak gradually disappears during discharge and reappears after charge in the in situ Raman spectra of the MnO_2_ slurry (Fig. 4a) [54–56]. Furthermore, the MnO_2_ slurry cell exhibits faster attenuation in Mn–O peak intensity than the CE-MnO_2_ cells during discharge owing to the rapid kinetic reaction in the 3D CNTs network (Fig. S36), indicating the superiority of the MnO_2_ slurry cell. However, there is always a Mn–O peak in the CE-MnO_2_ cells with and without AEM during charging and discharging (Figs. 4b, and S37), which results from incomplete MnO_2_ dissolution. Insight into the local dissolution being sought, we conducted MnO_2_ deposition on carbon paper in the CE-MnO_2_ cells. Dispersed void spaces are observed in SEM images of the MnO_2_ dissolution layer on carbon paper and gradually amplified as the discharge proceeds (Fig. S38). In situ optical observation allows direct visualization of MnO_2_ dissolution with local separation (Fig. S39), suggesting preferential dissolution of highly active regions on the MnO_2_ layer. Electrochemical impedance spectroscopy was employed to monitor in real time the changes in the MnO_2_ interface with increasing deposition thickness in a three-electrode device (Fig. S40). The interfacial reaction resistance increases with increasing MnO_2_ deposition capacity. It can be inferred that MnO_2_ preferentially dissolves at the thinner regions on the deposited MnO_2_ layer and extends along the transverse direction with increasing dissolution time. Aggregative growth of MnO_2_ in the slurry was investigated by X-CT since the formation of block deposits in semisolid slurries can significantly hinder ion transport [57]. As shown in Fig. 4c, d, MnO_2_ sedimentation with aggregation growth in the slurry cathode is effectively evaded owing to the stress-supported CNTs network with uniform 3D connected conduction and deposited γ-MnO_2_ with high electronic conductivity. MnO_2_ deposition on the CC and CNTs was simulated by FEA methods (Fig. S41). For the same deposition capacity, the thickness of the MnO_2_ layer deposited at micron-diameter carbon fibers on CC with finite surface areas is much higher than that deposited on the dispersed CNTs (Fig. 4e, f).

### Mechanism Studies of MnO_2_ Structure Evolution in the Slurry Electrode

To further elucidate the structural evolution of electrodeposited MnO_2_, density functional theory (DFT) calculations were conducted to study the interfacial interactions of Mn species and water (H_2_O) molecules with different carbon substrates (Figs. 4g and S42). As shown in Fig. 4h, the binding energy of Mn on CNTs (− 3.01 eV) is significantly lower than that on CC (− 4.87 eV). Also, the interaction between H_2_O and CNTs (− 0.11 eV) is weaker than that with CC (− 0.57 eV), suggesting that substrates with strong affinity for Mn and H_2_O tend to stabilize ε-MnO_2_, while CNTs suppress such interactions to promote γ-MnO_2_ formation. Interface energy analysis further supports this conclusion. The interfacial energy of ε-MnO_2_ on CC (− 0.27 eV Å^− 2^) is lower than that of γ-MnO_2_ (− 0.50 eV Å^− 2^), whereas on CNTs, γ-MnO_2_ (− 0.37 eV Å^− 2^) exhibits significantly lower energy than ε-MnO_2_ (− 0.95 eV Å^− 2^) (Fig. 4i). In situ electrochemical digital holography (EDH) was employed to monitor Mn^2+^ concentration at the electrode–electrolyte interface during MnO_2_ deposition and dissolution (Fig. 4j). Noticeable color changes were observed on CC–electrolyte interface during Mn^2+^ oxidation and MnO_2_ reduction, indicative of severe concentration polarization and inhomogeneous ion distribution. In contrast, the CNTs–electrolyte interface exhibits minimal concentration fluctuations, attributed to the rapid ion diffusion facilitated by the high active surface area of CNTs. As calculated from linear polarization curves, an interface exchange current density at CC is 3.05 × 10^− 3^ A cm^− 2^ (Fig. S43), higher than that at CNTs (6.67 × 10^− 4^ A cm^− 2^), consistent with calculation results. Therefore, the inhomogeneous ε-MnO_2_ formation on the CC in the CE-MnO_2_ cells is due to the strong Mn/H_2_O affinity of CC with high interface exchange current density. MnO_2_ selectively is dissolved in the thin regions of ε-MnO_2_ layer with uneven thickness (Fig. S44). However, weak interactions between Mn/H_2_O and CNTs, along with low exchange current density on the CNTs surface, promote the formation of a uniform and highly conductive γ-MnO_2_ thin layer. As a result, overall dissolution is achieved on the γ-MnO_2_ layer of the MnO_2_ slurry, enabling enhanced MnO_2_ deposition/dissolution reversibility.

**Fig. 5:**
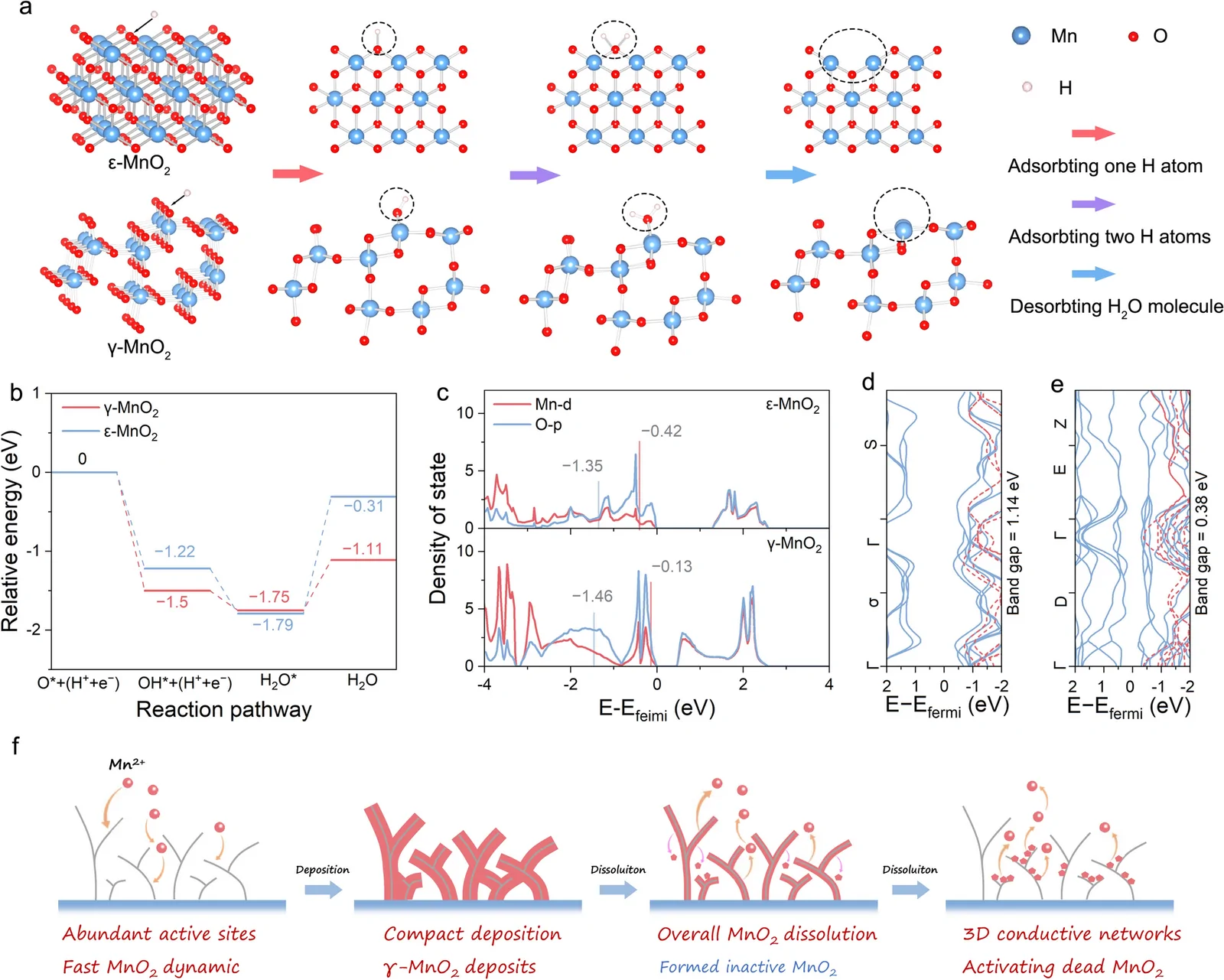
DFT calculation of MnO_2_ dissolution. **a** Structural models and **b** relative energy profiles of ε-MnO_2_ and γ-MnO_2_ dissolution with a separation of H_2_O. **c** PDOS of the O p-band and Mn d-band of ε-MnO_2_ and γ-MnO_2_ with band center values. Band structures of **d** ε-MnO_2_ and **e** γ-MnO_2_ with band gap values. **f** Schematic illustration of vitalizing inactive MnO_2_ formed during dissolution via the slurry electrode

The dissolution mechanisms of ε-MnO_2_ and γ-MnO_2_ were further analyzed by DFT calculations (Fig. 5a). The dissolution of MnO_2_ involves the interaction of protons (H) in the electrolyte with lattice oxygen (O) in MnO_2_ to form H_2_O molecules. The first H attacks the O on the MnO_2_ surface to form the OH* intermediate. Then, the second adsorbed H reacts with the resulting OH* to produce H_2_O, which is desorbed from the MnO_2_ surface. The H adsorption reaction is spontaneous on both ε-MnO_2_ and γ-MnO_2_ surfaces (Fig. 5b). However, an energy of 1.48 eV is required for the formed H_2_O to be desorbed from the ε-MnO_2_ surface. By contrast, the separation of H_2_O from the γ-MnO_2_ surface requires only overcoming an energy barrier of 0.64 eV. Partial density of states (PDOS) analysis in Fig. 5c shows that the Mn d-band center of γ-MnO_2_ is close to the Fermi energy level in comparison with that of ε-MnO_2_, which is favorable for high charge delocalization and active electronic states. Besides, the energy band gap of γ-MnO_2_ is 0.38 eV, significantly lower than that of ε-MnO_2_ (1.14 eV) (Fig. 5d, e). This result demonstrates that the formed conduction γ-MnO_2_ phase promotes fast reaction kinetics and charge transfer, thus catalyzing the electrolysis kinetics with the overall dissolution. Based on theoretical simulations and experimental analyses, MnO_2_ deposition/dissolution in slurry electrodes can be systematically elucidated in Fig. 5f. First, the 3D connected CNTs networks with weak Mn/H_2_O interaction provide abundant highly active sites for MnO_2_ deposition and fast electron transport, leading to the generation of dense conductive γ-MnO_2_ deposits with a robust interface between γ-MnO_2_ and CNTs. In the initial step of dissolution, uniform MnO_2_ dissolves from the CNTs into the slurry electrolyte with the inevitable separation of MnO_2_ solids to form inert MnO_2_. However, those electrochemical inert MnO_2_ can connect with adjacent CNTs and regain electrons, thereby redissolving into the electrolyte as discharging progresses.

## Conclusions

In summary, we develop MnO_2_ deposition/dissolution chemistry in a semisolid slurry by uniformly dispersing commercial CNTs into the electrolyte for advanced electrolytic Zn–MnO_2_ batteries. The electron-percolating network constructed by the CNTs significantly enhances charge transport and facilitates the formation of highly conductive γ-MnO_2_ deposits, promoting overall MnO_2_ dissolution instead of localized degradation. Without the employment of binders, the slurry electrode system allows facile separation and regeneration, contributing to extended cycling life. The MnO_2_ slurry exhibits a discharge capacity close to 60 mAh cm^− 2^ under a flow cell configuration, effectively overcoming the reactive area constraints of conventional carbon-based electrodes. Besides, the 3D conducting CNTs rheology system renders the MnO_2_ slurry with inactive manganese revitalization. An important direction for future investigation will be the in-depth study of the slurry’s rheological properties to further optimize long-duration energy storage performance. We expect this work to serve as a foundation for the development of novel electrode systems and functional components in next-generation energy storage technologies.

## Supplementary Information

Below is the link to the electronic supplementary material.

Supplementary file 1 (DOCX 15501 KB)
