# Supplementary material for: Unlocking Reversible Mn2+/MnO2 Chemistry in Semisolid Slurry Electrodes for High-Performance Aqueous Zn–Mn Batteries
Source: Nanomicro Lett. 2026 Jan 12;18:148. doi: 10.1007/s40820-025-01994-9 (PMC12791095; doi:10.1007/s40820-025-01994-9)
Supplement: Supplementary file 1 — (DOCX 15501 KB) [file 40820_2025_1994_MOESM1_ESM.docx]

Supporting Information for

**Unlocking Reversible Mn^2+^/MnO_2_ Chemistry in Semisolid Slurry Electrodes for High-Performance Aqueous Zn-Mn Batteries**

Zefang Yang^1,2,3^, Qi Zhang^1^*, Chao Hu^1^, Yougen Tang^1^, Jinchi Li^2^, Qi Wang^4^, Wanhai Zhou^2^, Dongliang Chao^2^*, Haiyan Wang^1^*

^1^ Hunan Provincial Key Laboratory of Chemical Power Sources, College of Chemistry and Chemical Engineering, Central South University, Changsha 410083, P. R. China

^2^ Laboratory of Advanced Materials, Aqueous Battery Center, State Key Laboratory of Molecular Engineering of Polymers, College of Smart Materials and Future Energy, Fudan University, Shanghai 200433, P. R. China

^3^ School of Physical and Mathematical Sciences, Nanyang Technological University, Singapore 637371, Singapore

^4^ Nanotechnology Research Laboratory, Faculty of Engineering, University of Sydney, NSW 2006, Australia

*Corresponding authors. E-mail: [qzhang1027@csu.edu.cn](mailto:qzhang1027@csu.edu.cn) (Qi Zhang); [chaod@fudan.edu.cn](mailto:chaod@fudan.edu.cn) (Dongliang Chao); [wanghy419@csu.edu.cn](mailto:wanghy419@csu.edu.cn) (Haiyan Wang)

**Supplementary Figures**


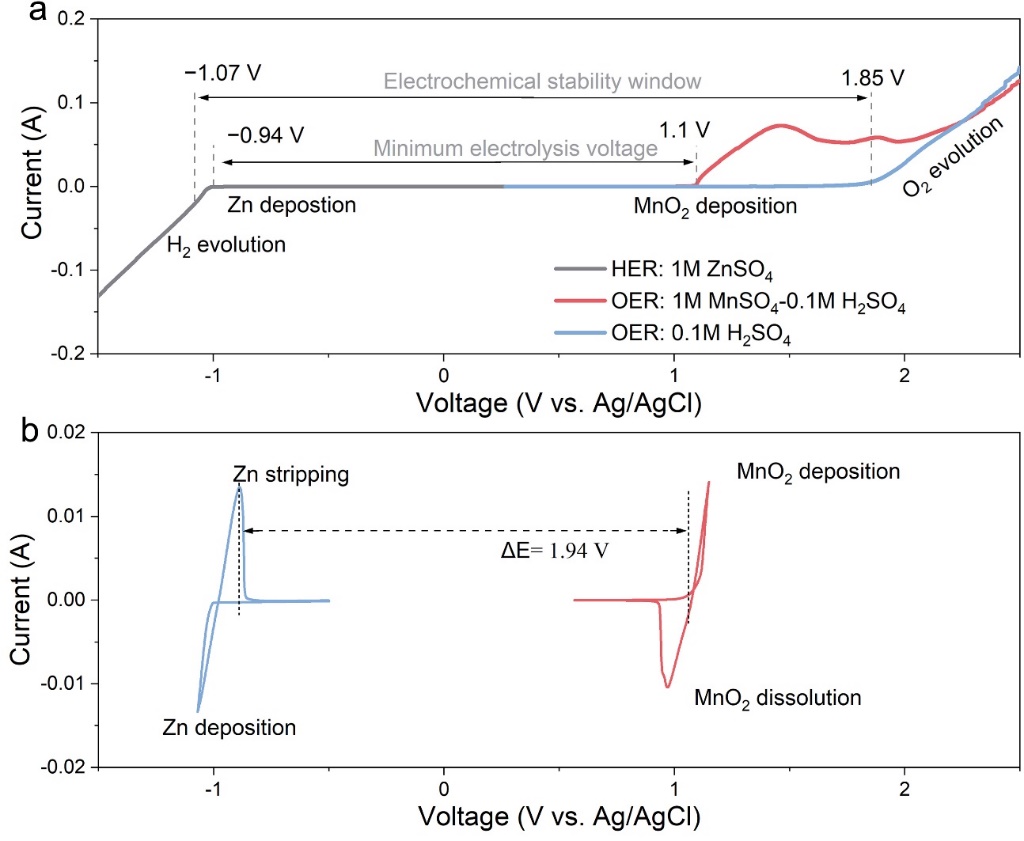


**Fig. S1** (**a**) Cyclic voltammetry curve of MnO_2_ and zinc deposition/dissolution with a voltage gap. (**b**) The electrochemical window of the electrolyte for the slurry electrode


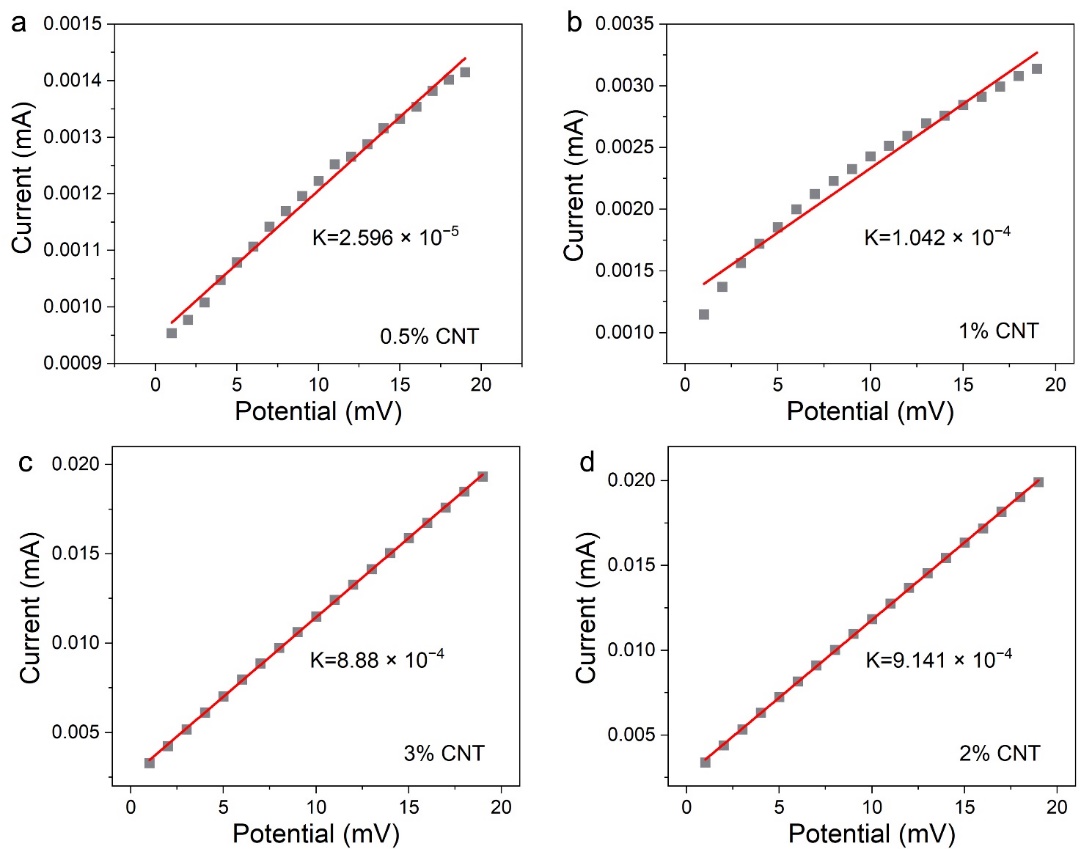


**Fig. S**2 Voltage-current relationship of the slurry with CNTs concentrations of (**a**) 0.5%, (**b**) 1%, (**c**) 2% and (**d**) 3%

The resistance of the slurry was calculated using the previous methods. [S1, S2] The electrode slurry is first encapsulated between the graphite plates. The measurement is conducted on the electrochemical workstation at a fixed scan rate of 0.1 mV s^−1^. The electronic conductivity of slurry can be calculated using the formula below.

*R* = *U*/*I* (S1)

*σ* = *L*/*RS* (S2)

where *R*, *L* and *S* are the slope (1/*K*) of the voltage-current plot, the thickness of the slurry electrode and the reaction area of the slurry, respectively.


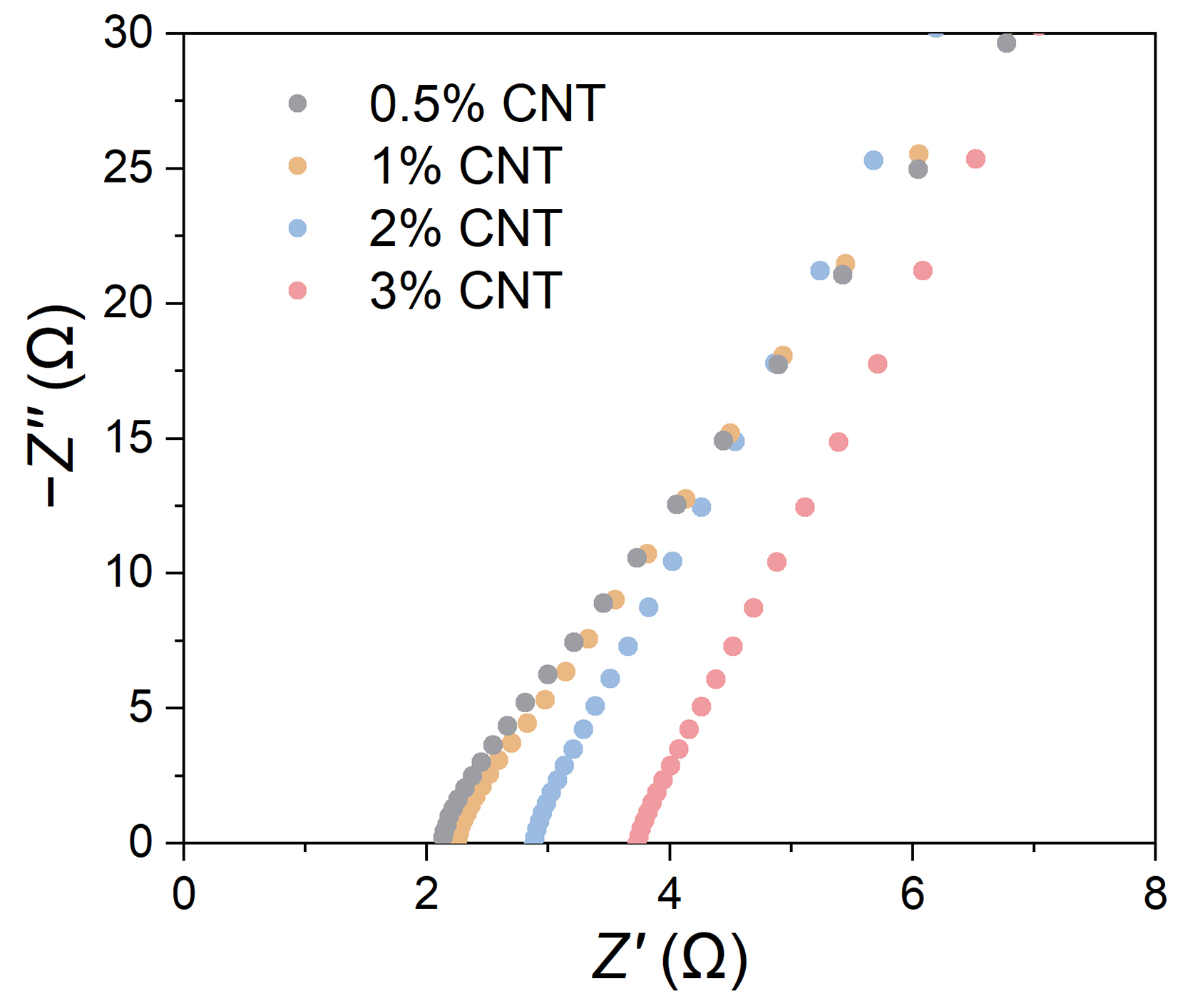


**Fig. S3** Nyquist plots of the slurry with different concentrations of CNTs. The measurements are conducted by employing two stainless sheets as work and counter electrodes, respectively


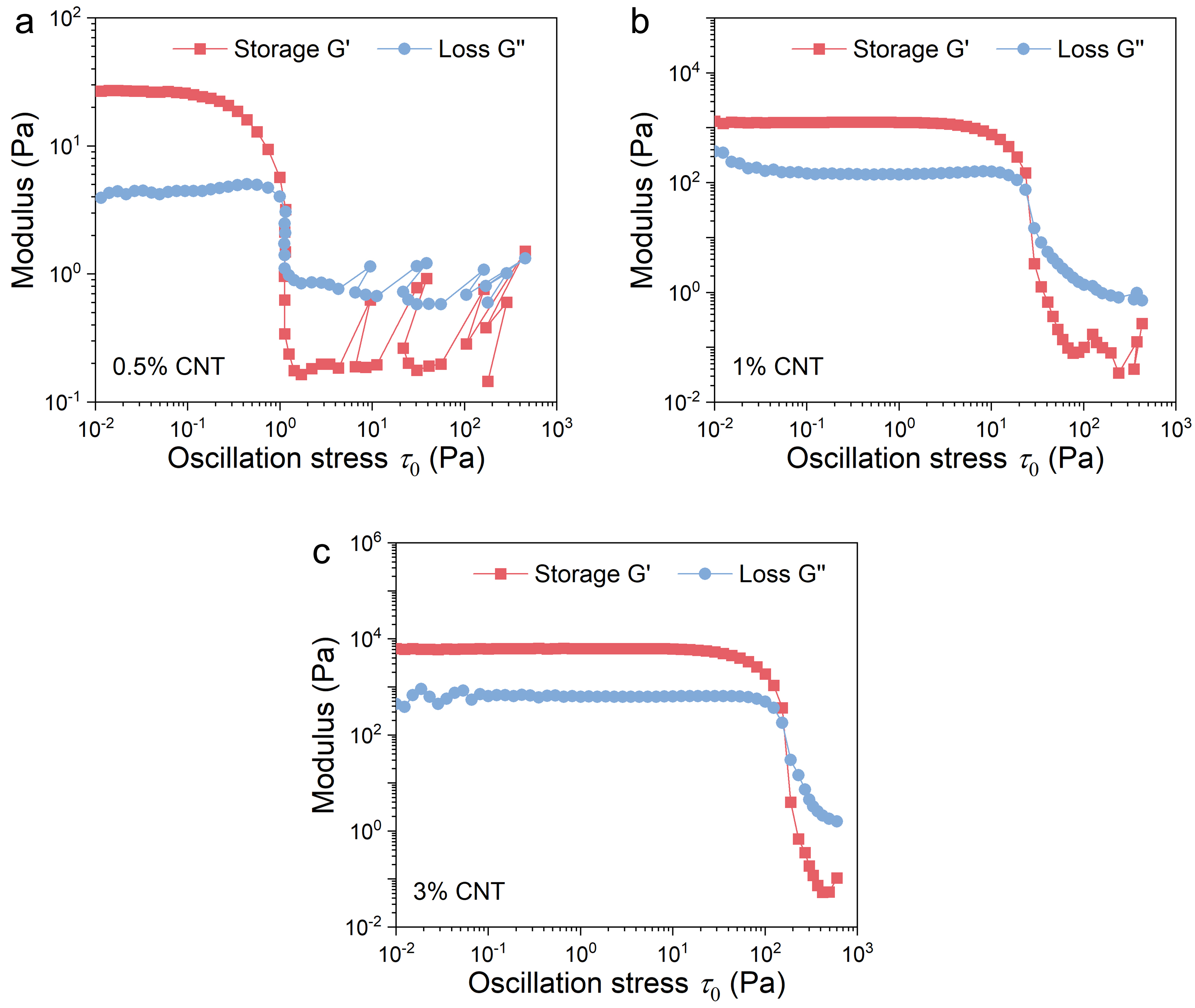


**Fig. S4** Rheological property of the slurry with a CNTs concentration of (**a**) 0.5%, (b) 1% and (**c**) 3%


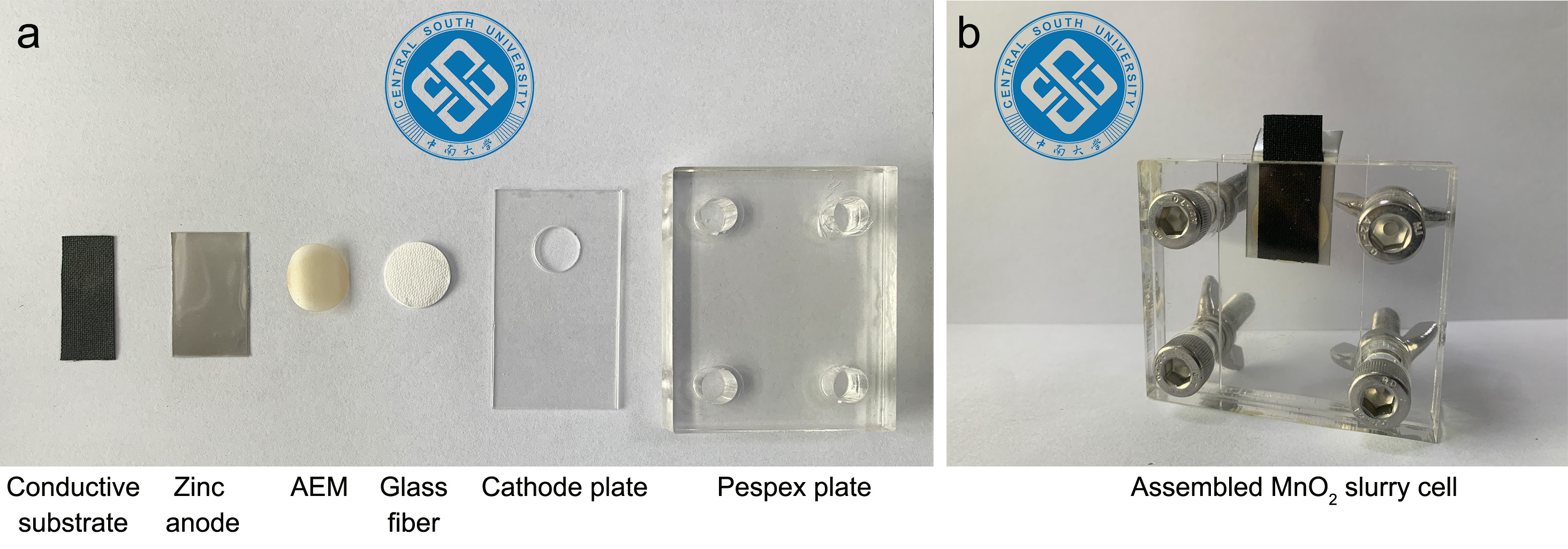


**Fig. S5** (**a**) The units and (**b**) setup of the Perspex-type MnO_2_ slurry cell


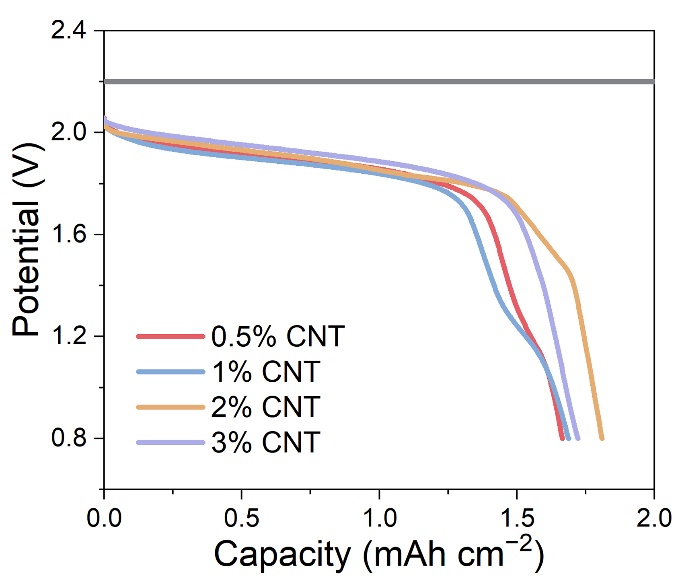


**Fig. S6** The constant-voltage charge and galvanostatic discharge curves of the slurry electrodes with different concentrations of CNTs


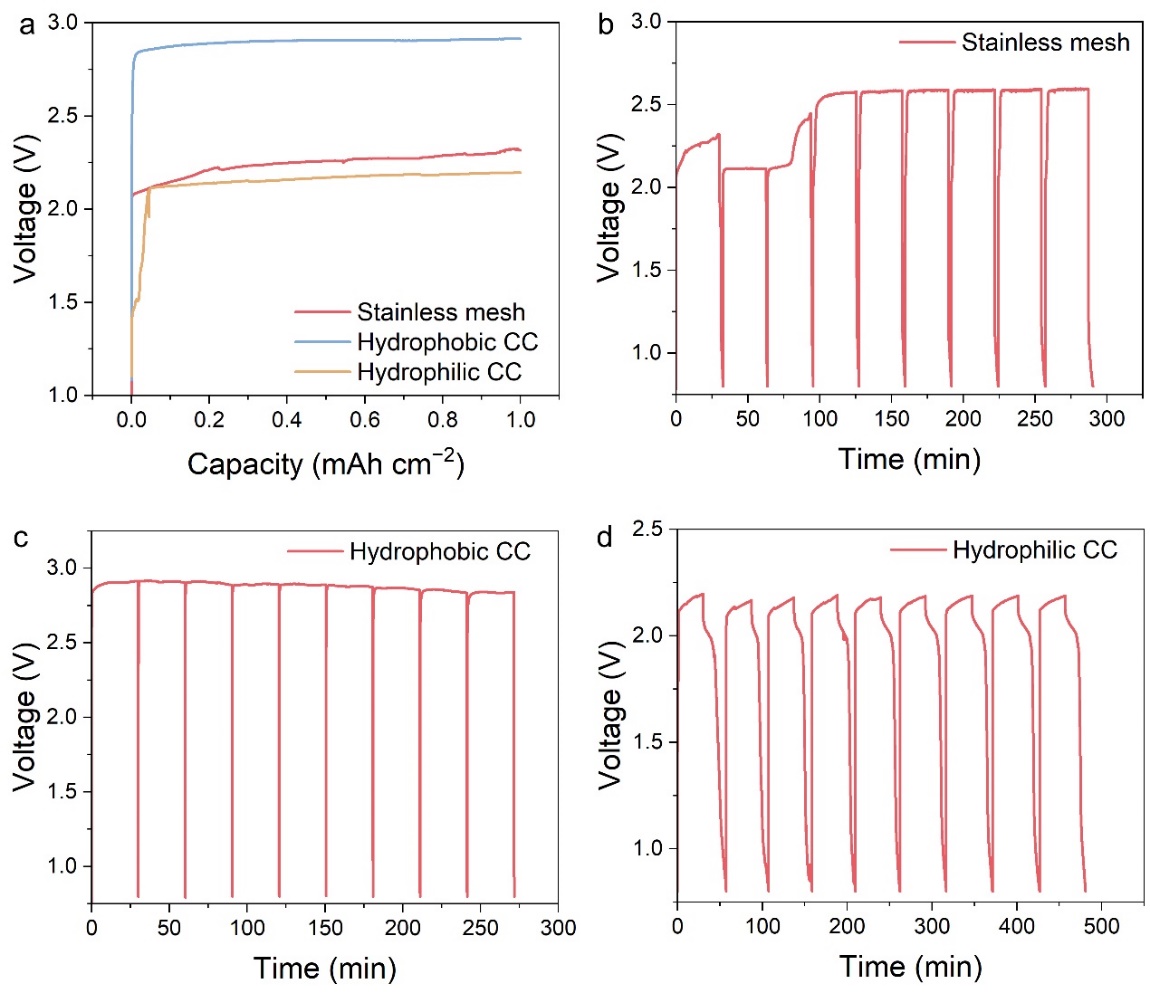


**Fig. S7** (**a**) Voltage curves of MnO_2_ deposition on stainless mesh, hydrophobic and hydrophilic CC in the catholyte at a constant current. Cycling curves of MnO_2_ deposition/dissolution on (**b**) stainless mesh, (**c**) hydrophobic and (**d**) hydrophilic CC


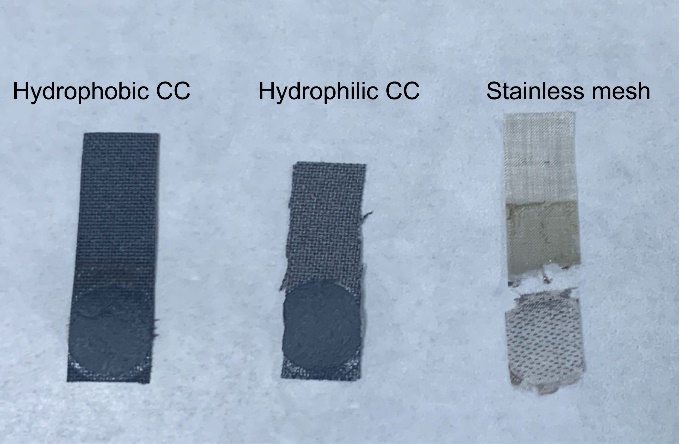


**Fig. S8** Digital photographs of stainless mesh, hydrophobic and hydrophilic CC disassembled from electrolytic MnO_2_ cell after cycling

MnO_2_ deposition/dissolution on stainless mesh, hydrophobic and hydrophilic CC was investigated. Hydrophobic CC exhibits a high MnO_2_ deposition overpotential close to electrolytic water, whereas the MnO_2_ deposition overpotentials on hydrophilic CC and stainless mesh are 2.19 and 2.32 V, respectively. It can be seen from the voltage profiles of MnO_2_ on hydrophobic CC that no MnO_2_ dissolution behavior occurs during the cycling period. In addition, only a tiny amount of MnO_2_ is separated from the stainless mesh. The fracture on the stainless mesh is observed after repeated MnO_2_ deposition/dissolution. Both hydrophilic and hydrophobic CC show no change in integrity and appearance. Note that the current collector for the slurry is supposed to be highly conductive to rapidly transport electrons but inert to MnO_2_ to ensure reversible MnO_2_ deposition/dissolution on the CNTs in the slurry. Therefore, an optimized hydrophobic CC is chosen as the conductive substrate for the slurry.


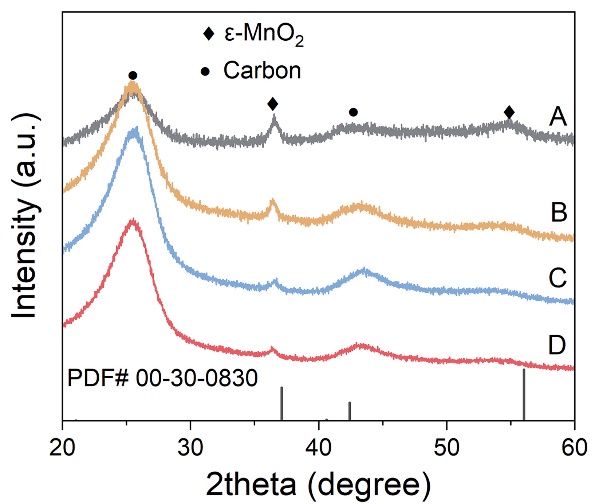


**Fig. S9** XRD patterns of MnO_2_ deposition/dissolution in CE-MnO_2_ cells with AEM


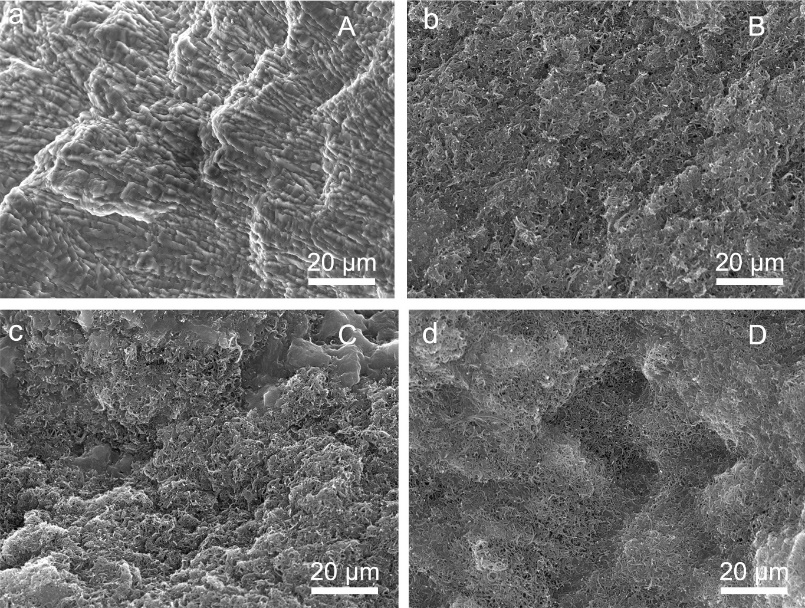


**Fig. S10** SEM images of MnO_2_ slurry electrode after (**a**) the charge and (**b-d**) discharge at different states


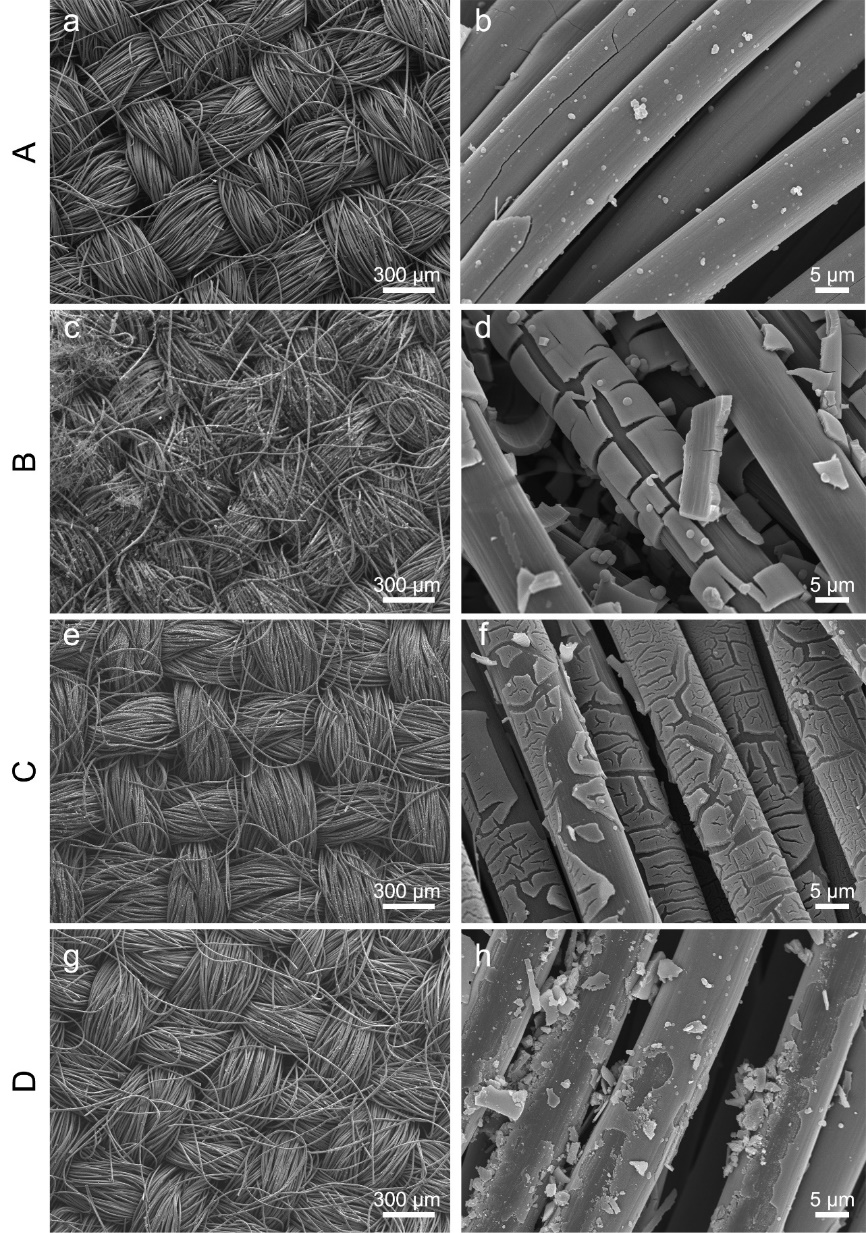


**Fig. S11** SEM images of CE-MnO_2_ cell without AEM after (**a, b**) the charge and (**c-h**) discharge at different states


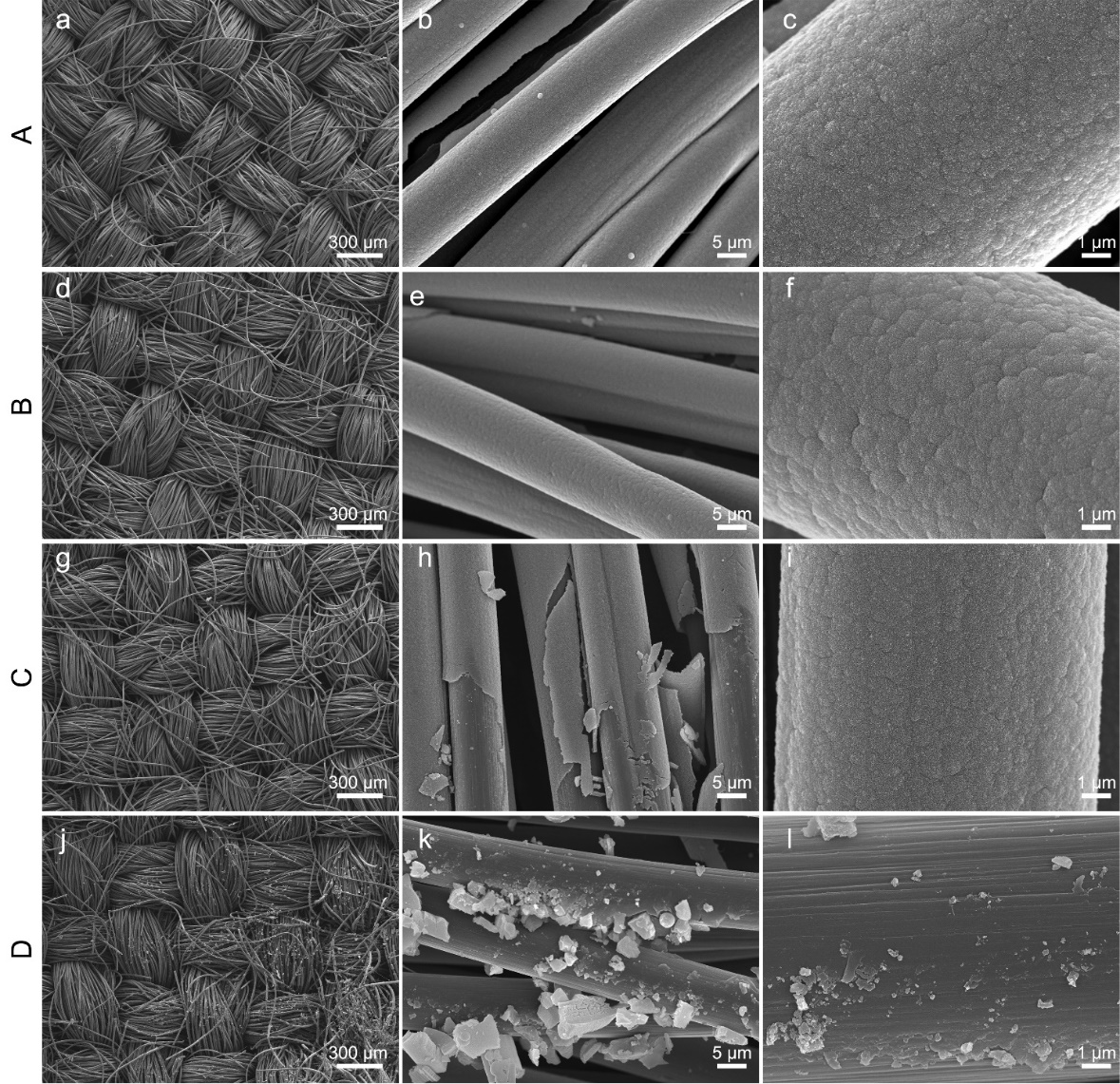


**Fig. S12** SEM images of CE-MnO_2_ cell with AEM after (**a-c**) the charge and (**d-l**) discharge at different states


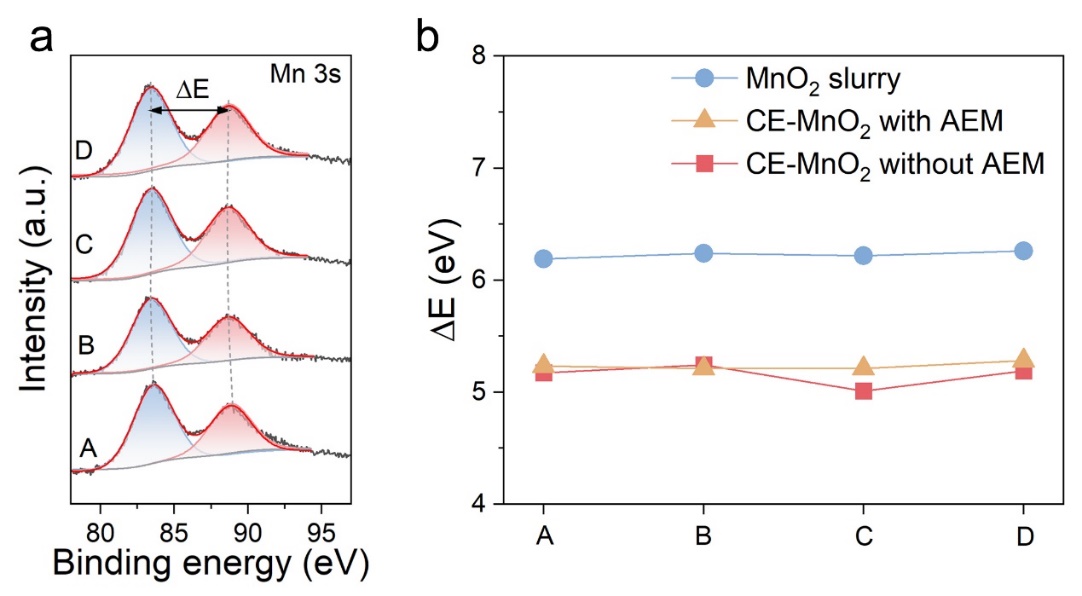


**Fig. S13** (**a**) Mn 3s spectra of CE-MnO_2_ cells with AEM. (**b**) ∆E value of MnO_2_ slurry and CE-MnO_2_ cells with and without AEM after charge and discharge at different states by single peak fitting


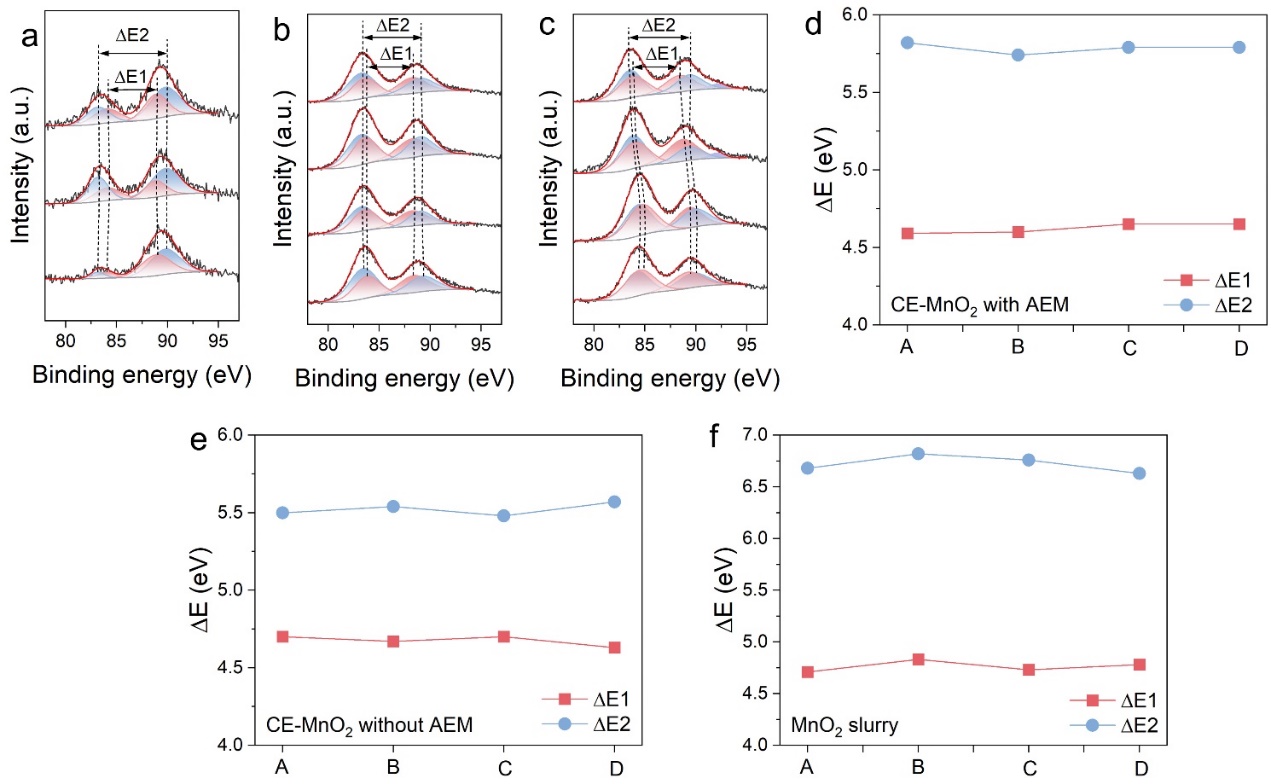


**Fig. S14** Mn 3s spectra of CE-MnO_2_ cells (**a**) with and (**b**) without AEM after charge and discharge at different states by multi-peak fitting. ∆E value of CE-MnO_2_ cells (**c**) with and (**d**) without AEM and (**e**) MnO_2_ slurry cell by multi-peak fitting


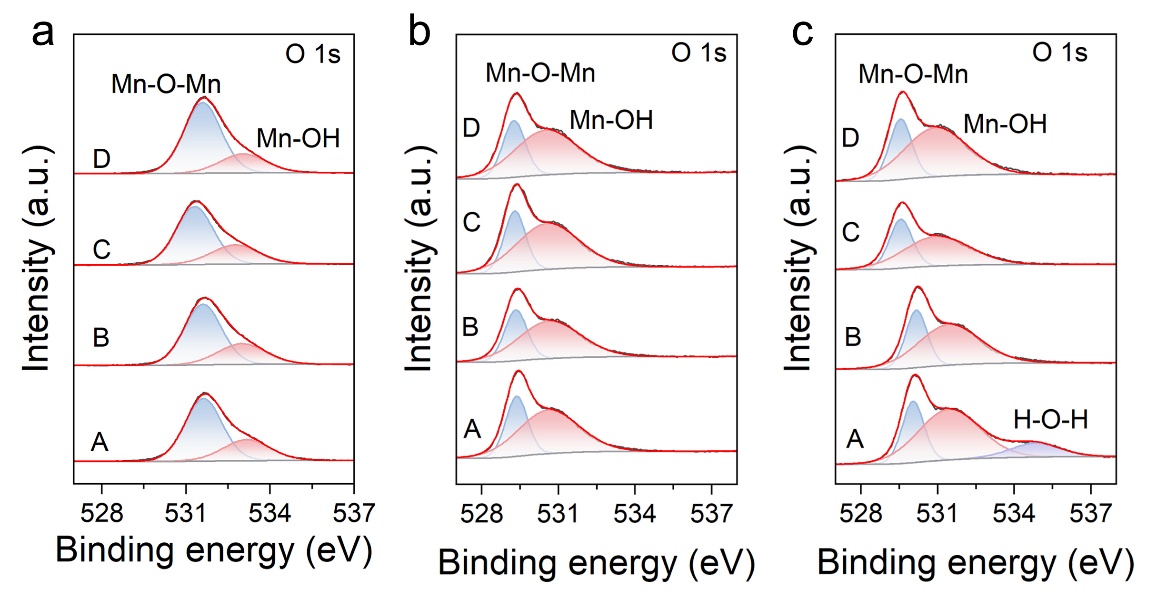


**Fig. S15** O 1s spectra of (**a**) the MnO_2_ slurry and CE-MnO_2_ cells (**b**) with and (**c**) without AEM after charge and discharge at different states


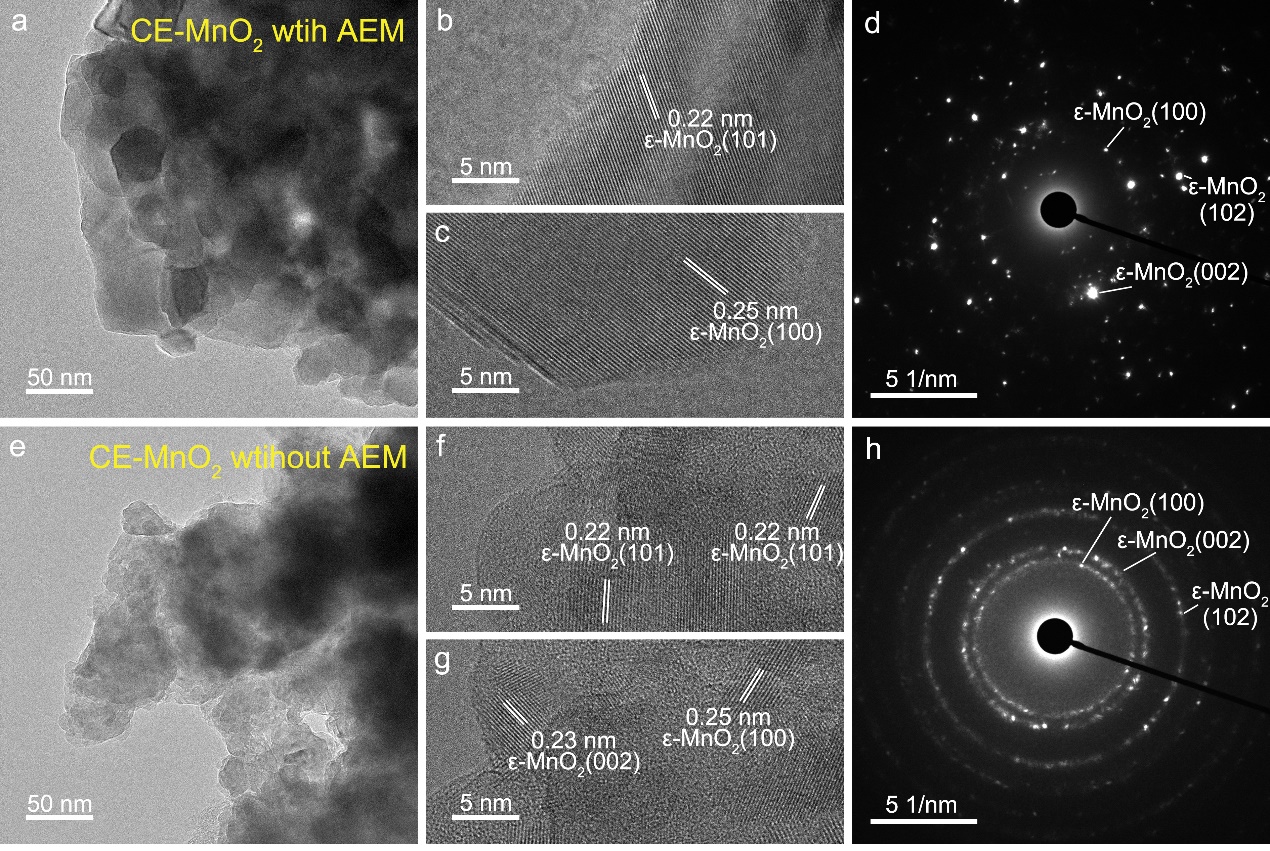


**Fig. S16** (**a, e**) TEM images, (**b, c, f, g**) high-resolution TEM images and (**d, h**) the selected area electron diffraction patterns of MnO_2_ deposition in CE-MnO_2_ cells (**a-d**) with and (**e-h**) without AEM


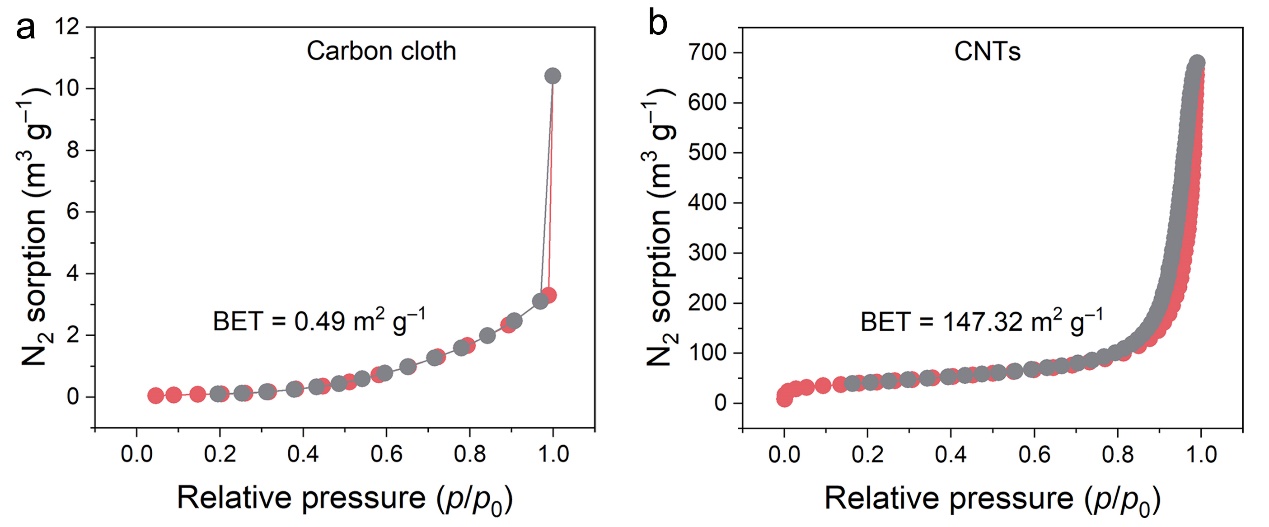


**Fig. S17** N_2_ adsorption–desorption isotherms of (**a**) carbon cloth and (**b**) CNTs


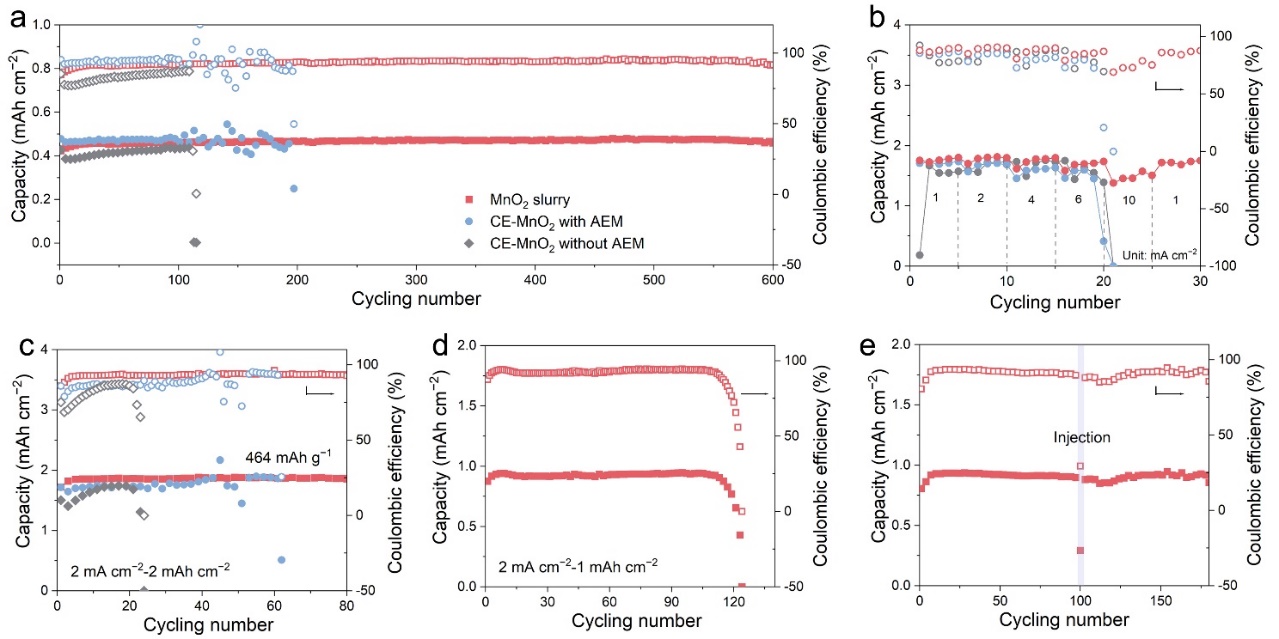


**Fig. S18** (**a**) Cycling stability and (**b**) rate performance of Zn-MnO_2_ slurry and CE-MnO_2_ cells with and without AEM. (**c**) Cycling performance of Zn-MnO_2_ slurry and CE-MnO_2_ cells with and without AEM at 2 mA cm^−2^ and 2 mAh cm^−2^. (**d**) Cycling performance of Zn-MnO_2_ slurry cells at 2 mA cm^−2^ and 1 mAh cm^−2^. (**e**)The cycling life of the Zn-MnO_2_ slurry cell was enhanced by the injection of the catholyte

The charge and discharge conditions are further set to 2 mA cm^−2^ and 2 mAh cm^−2^. As shown in **Fig. S**18c, the MnO_2_ slurry cell delivers a specific capacity of 464 mAh g^−1^ (calculated based on the mass loading of active CNTs) and maintains a stable capacity after 80 cycles, whereas the capacity of CE-MnO_2_ cells with and without AEM is rapidly degraded after 44 and 21 cycles, respectively. Note that the thick MnO_2_ deposition layers are generated in the case of high mass loading, resulting in low dissolution reversibility due to the poor electronic conductivity of ε-MnO_2_. To investigate the failure mechanism of the MnO_2_ slurry cell, the cycling performance was tested at 2 mA cm^−2^ and 1 mAh cm^−2^. The MnO_2_ slurry cell shows a steady capacity profile within 110 cycles and a subsequent sharp capacity decline (**Fig. S**18d), which arises from the depletion of the electrolyte in the slurry by H_2_ evolution from the zinc anodes. Surprisingly, when the MnO_2_ slurry electrode is injected with a small amount of catholyte before failure, the cycle life of the slurry cells can be extended to 180 cycles, suggesting a low-cost regenerative capability of the slurry battery (**Fig. S**18e).


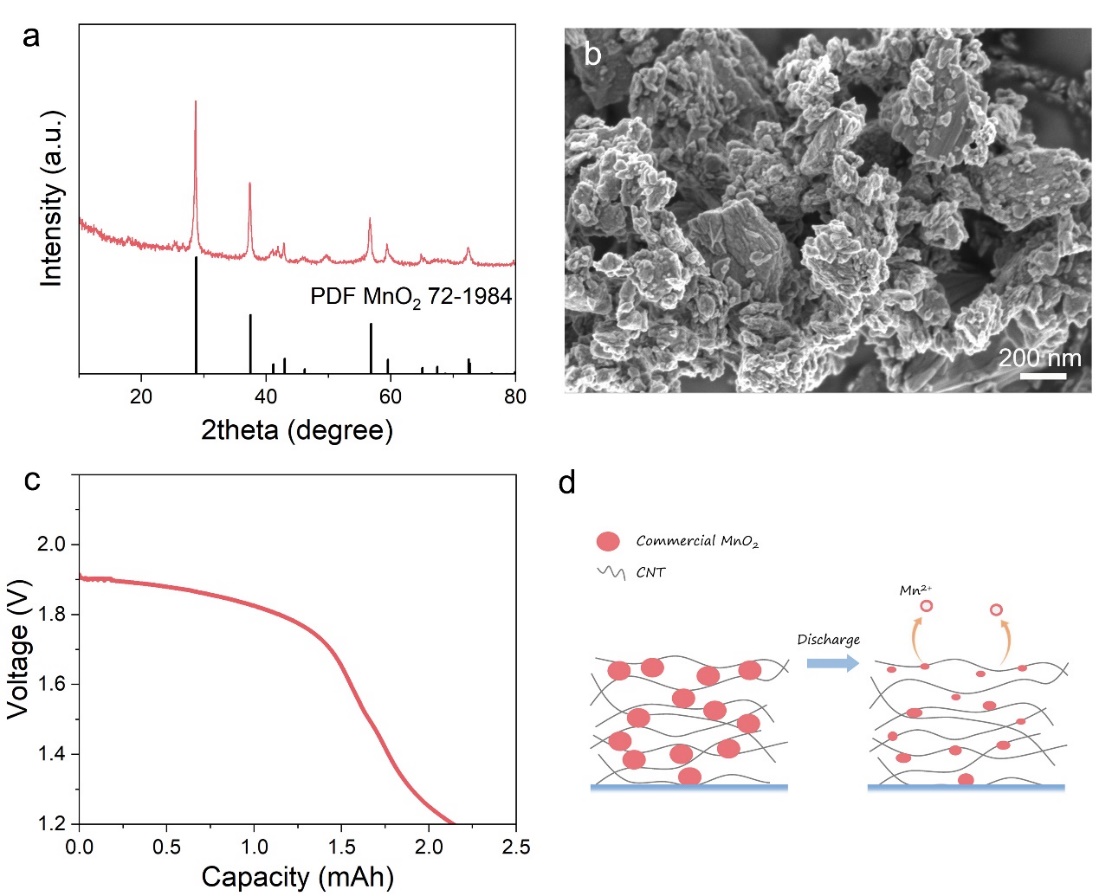


**Fig. S19** (**a**) XRD patterns and (**b**) SEM images of commercial MnO_2_ powder. (**c**) Discharge curves of slurry with appropriate amounts of commercial MnO_2_ powders. (**d**) Schematic illustration of commercial MnO_2_ powders being dissolved during discharge

Commercial MnO_2_ powders were added to the catholytes with the CNTs content of 2% to obtain a uniform slurry (**Fig. S**19a and S19b). When a galvanostatic discharge was applied to the slurry electrode with MnO_2_ powders, a distinct discharge curve with a voltage platform of 1.85 V was observed (**Fig. S**19c). The results indicate that the pre-incorporated MnO_2_ in the slurry can be activated by the 3D conductive CNTs network, suggesting the ability of the slurry electrode to revive dead MnO_2_ during MnO_2_ dissolution (**Fig. S**19d).


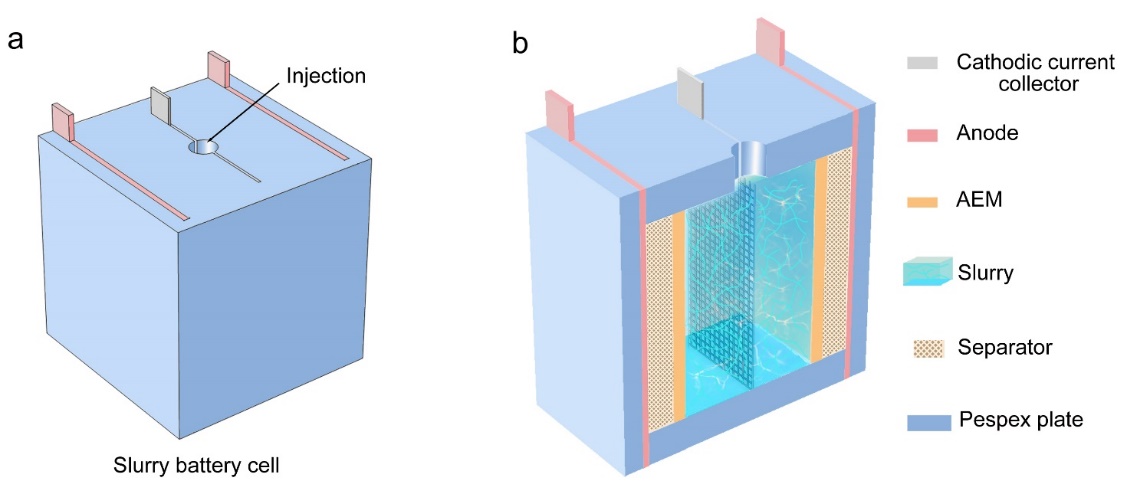


**Fig. S20** Schematic illustration of (**a**) appearance and (**b**) cross section for the Zn-MnO_2_ slurry cells


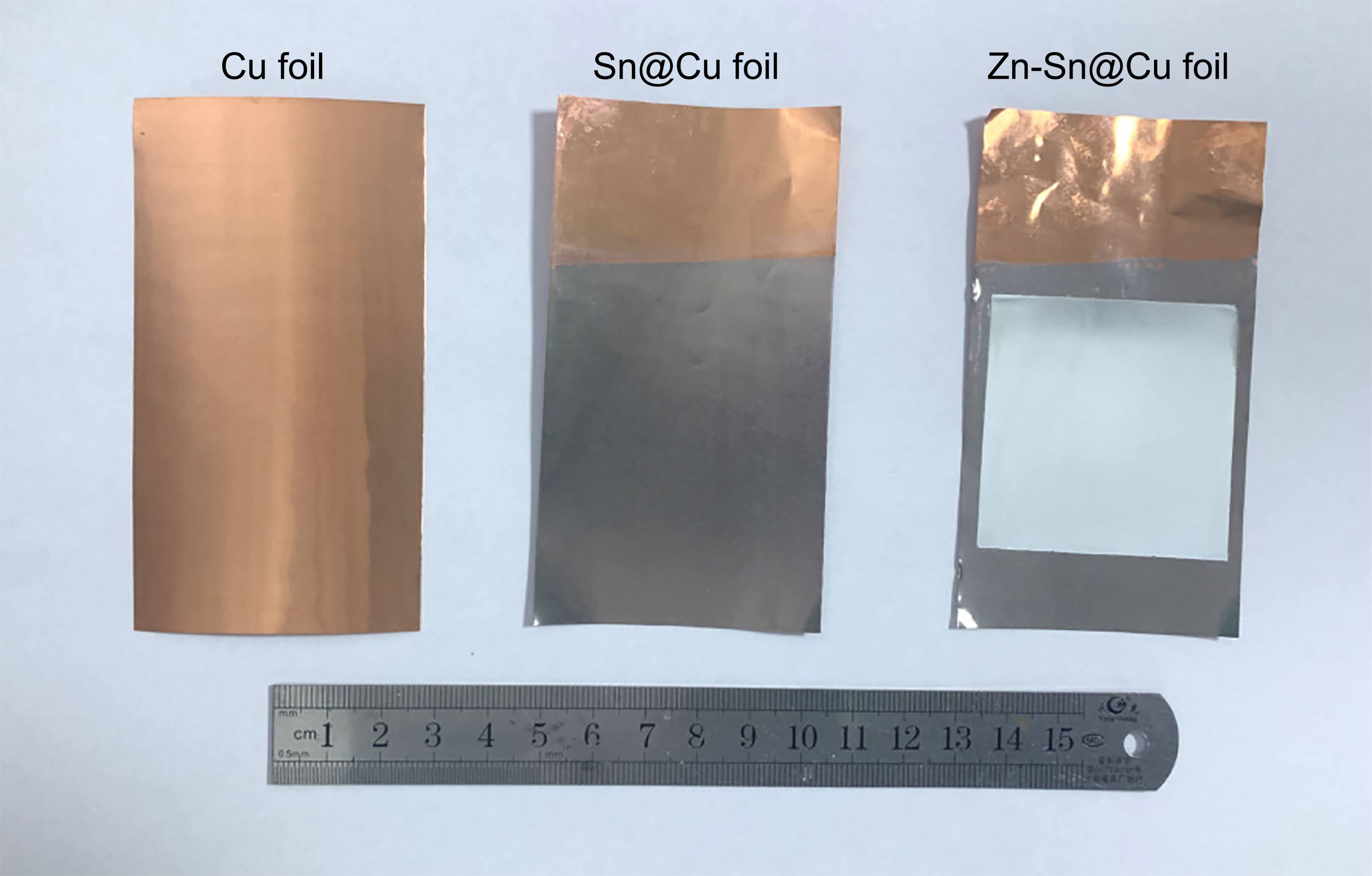


**Fig. S21** Digital photographs of copper (Cu) foil, tinned copper (Sn@Cu) foil and pre-plated zinc on Sn@Cu (Zn-Sn@Cu) foil


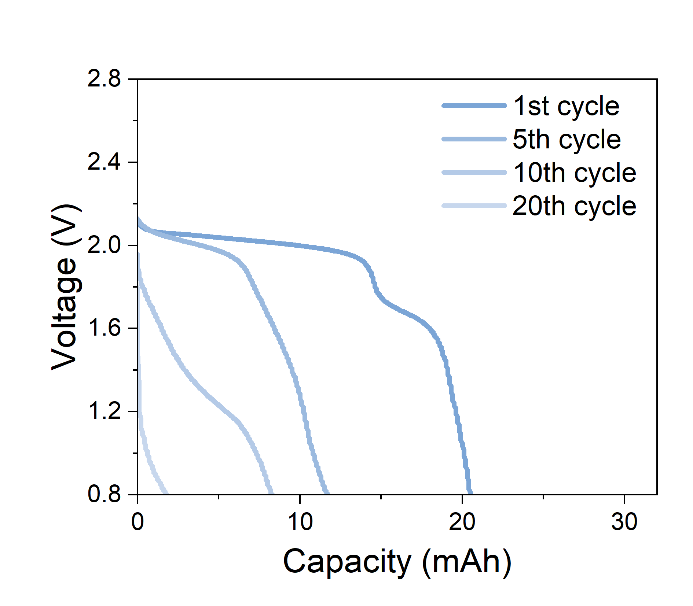


**Fig. S22** The discharge curves of CE-MnO_2_ battery with Zn-Sn@Cu anode


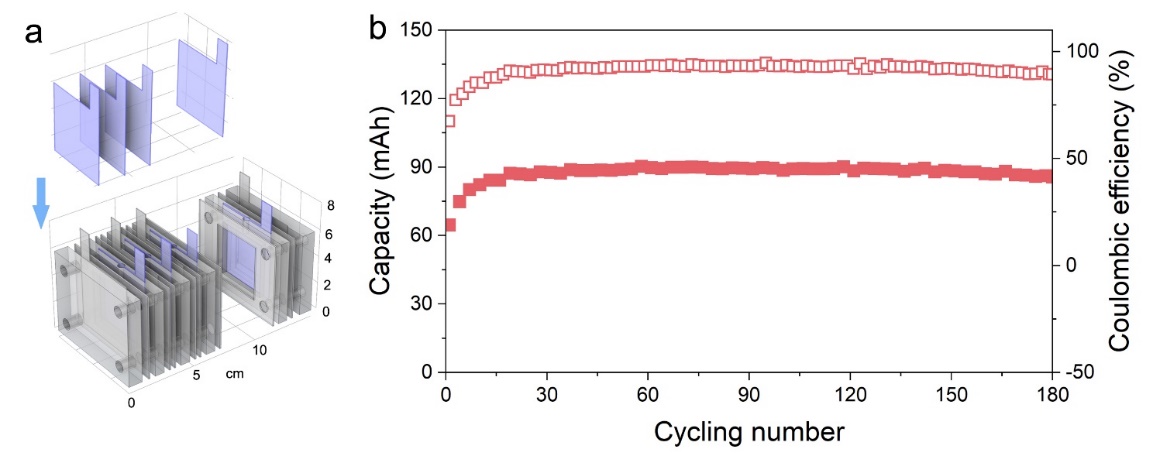


**Fig. S23** (**a**) Schematic illustration of fabrication for multi-layer slurry batteries with high voltage and capacity. (**b**) Cycling performance of Zn-MnO_2_ slurry battery with three slurry electrodes in parallel after charge for 96 mAh and discharge to 0.8 V at 384 mA


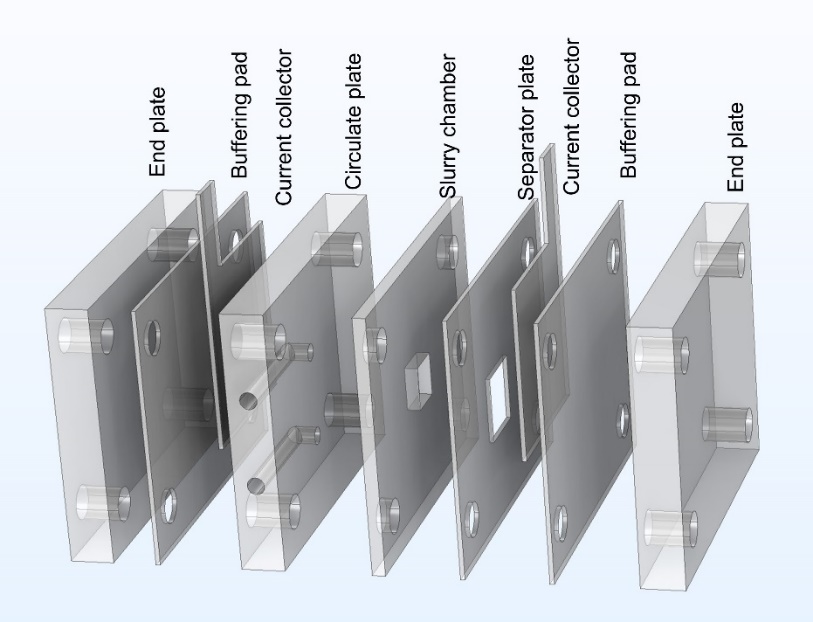


**Fig. S24** Schematic illustration of Zn-MnO_2_ slurry flow cell


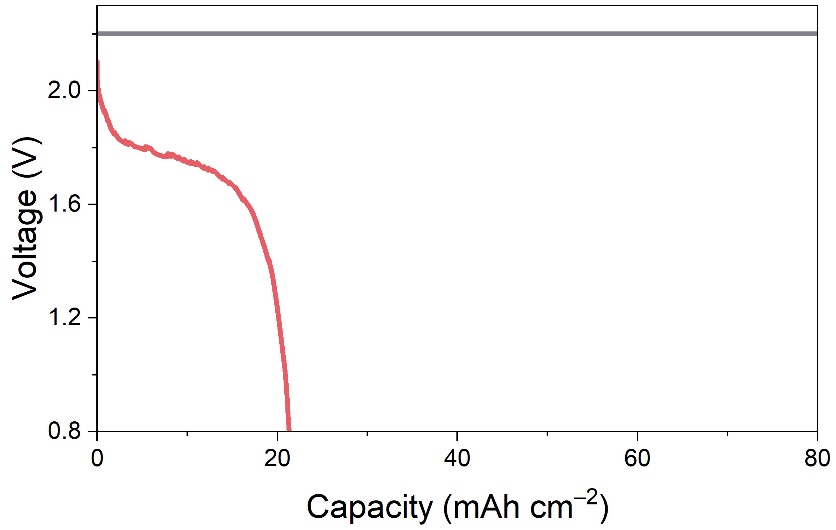


**Fig. S25** Charge and discharge curves of CE-MnO_2_ flow cell at a discharge current density of 1.5 mA cm^−2^


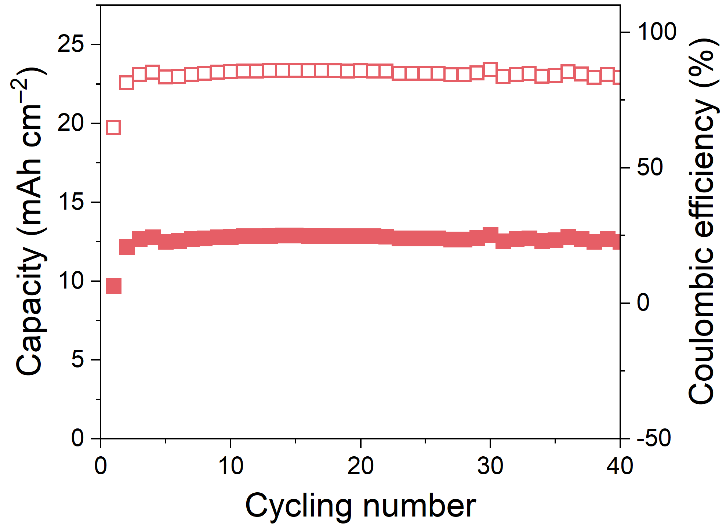


**Fig. S26** Cycling stability of the Zn-MnO_2_ slurry flow cell at 1.5 mA cm^−2^ and 15 mAh cm^−2^, a capacity of 12.5 mAh cm^−2^ is retained after 40 cycles with a CE of 83.3%


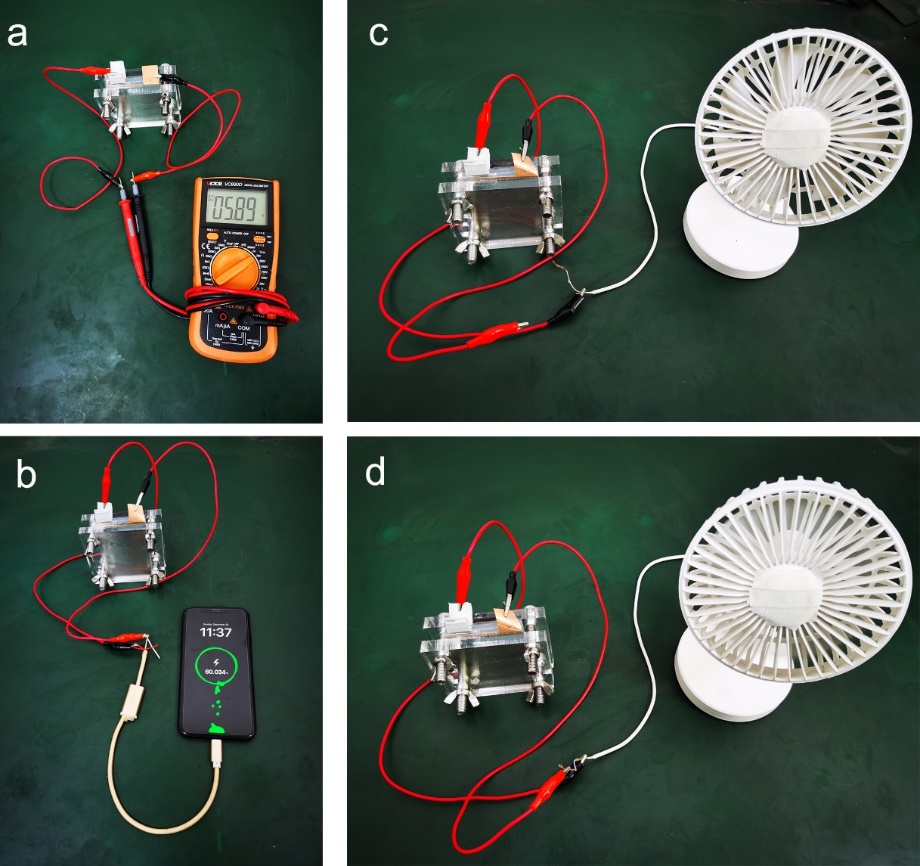


**Fig. S27** Digital photographs of (**a**) open-circuit voltage test, power supply for (**b**) mobile phone and (**c, d**) electric fan of the three Zn-MnO2 slurry cells connected in series; (**c**) disconnect state, (**d**) work state


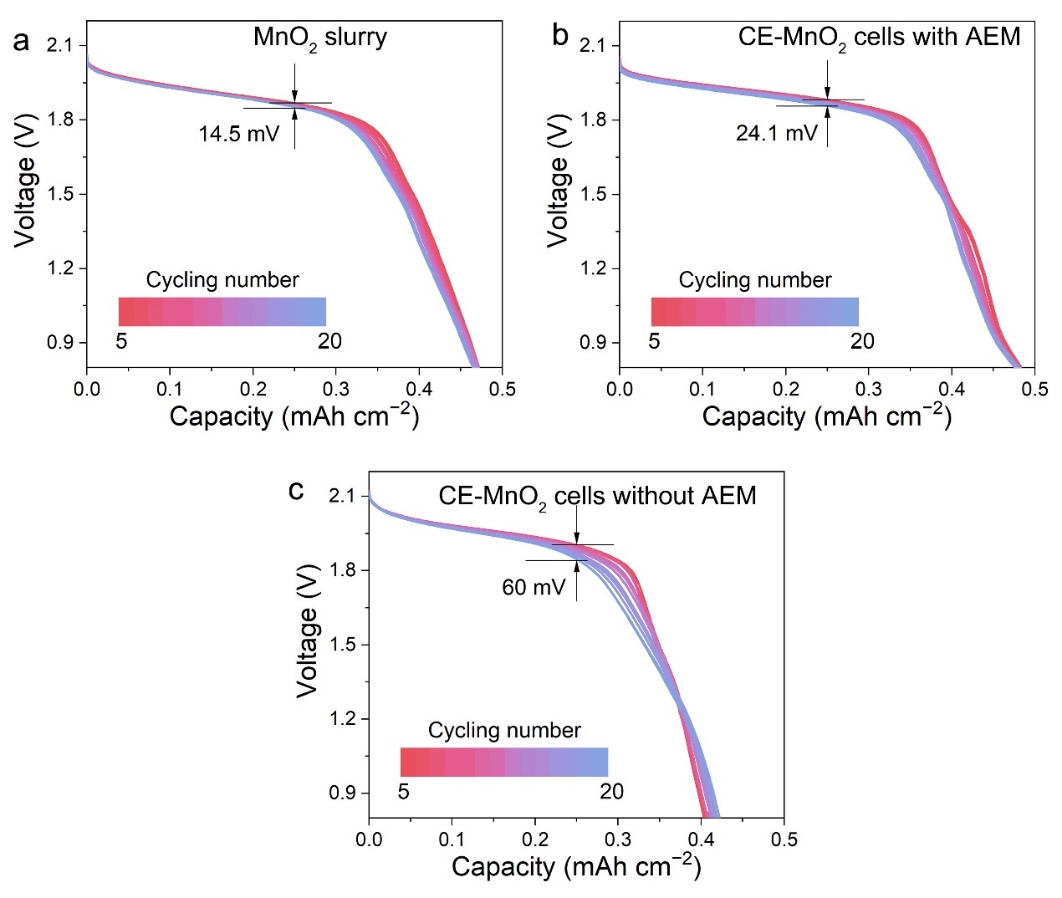


**Fig. S28** The discharge curves of (**a**) MnO_2_ slurry and CE-MnO_2_ cells (**b**) with and (**c**) without AEM in the range of 5th to 20th cycle


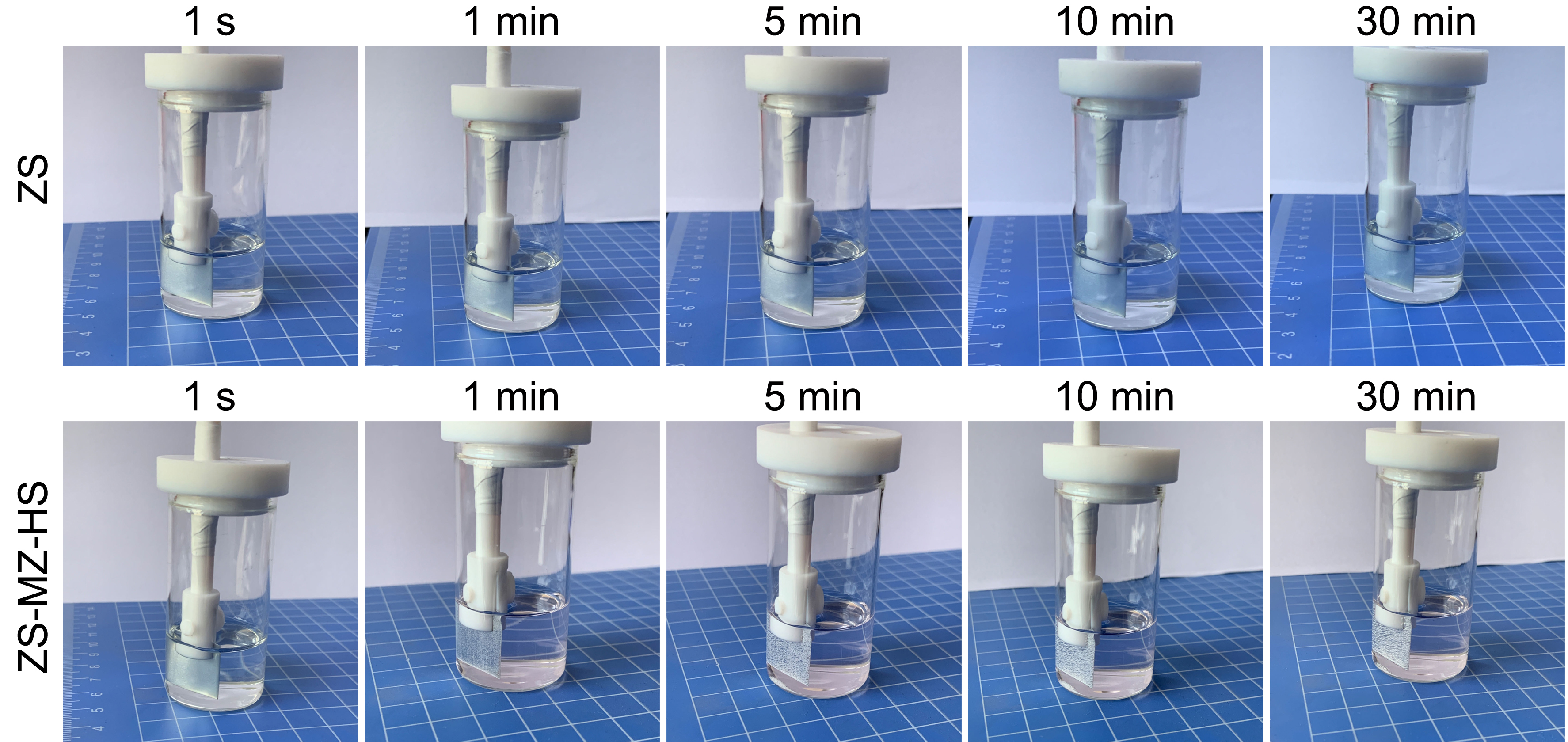


**Fig. S29** Digital photograph of zinc foil soaking in 1 M ZnSO_4_ (ZS) and 1 M MnSO_4_-1M ZnSO_4_ electrolytes with 0.1 M H_2_SO_4_ (ZS-MS-HS) additive for different time


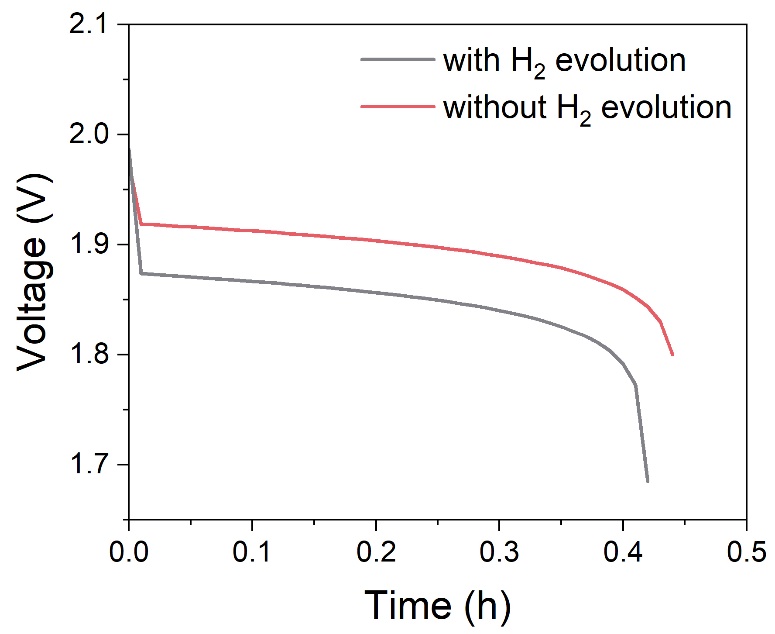


**Fig. S30** The simulated discharge curves of Zn-MnO_2_ cells with and without H_2_ evolution at the anode by FEA


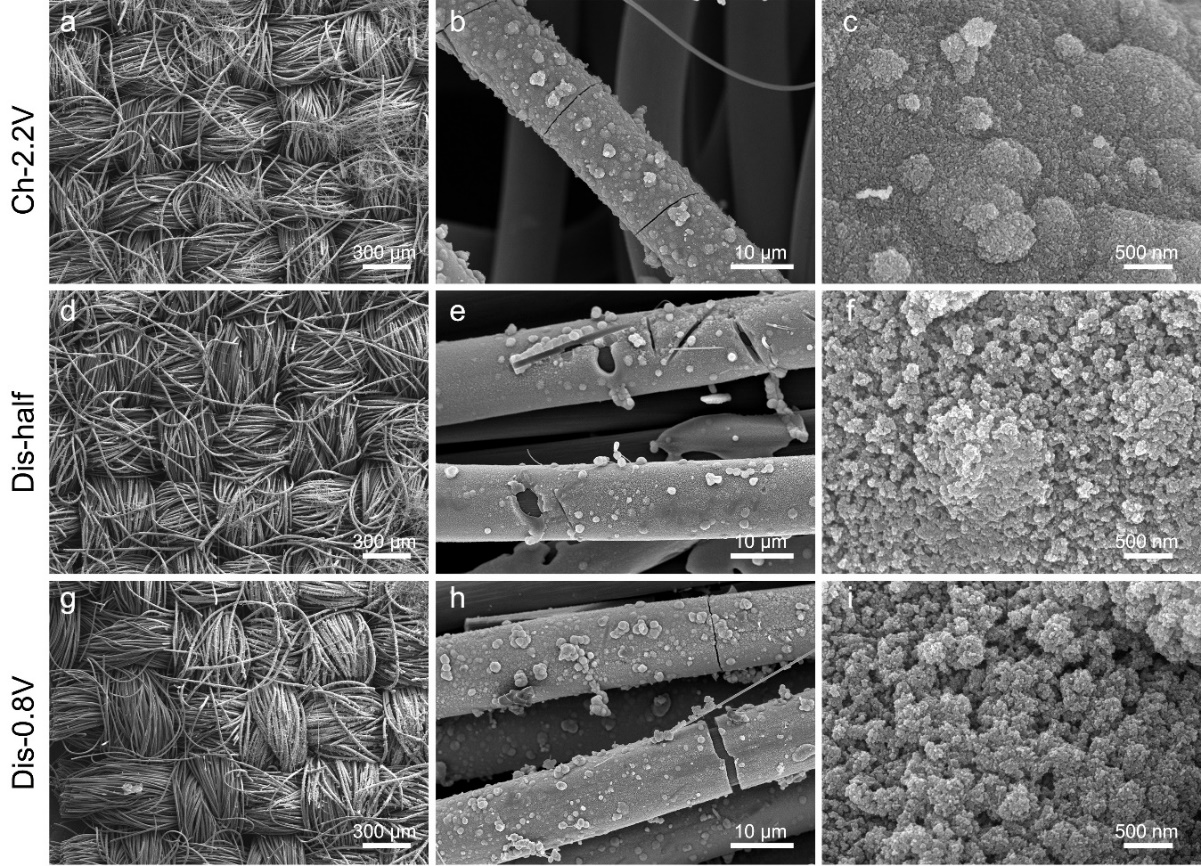


**Fig. S31** SEM images of the CE-MnO_2_ cell without AEM after 20 cycles at different states. (**a-c**) Ch-2.2V, (**d-f**) Dis-half and (**g-i**) Dis-0.8V represent full charge state, discharge to half depth of discharge and 0.8 V, respectively


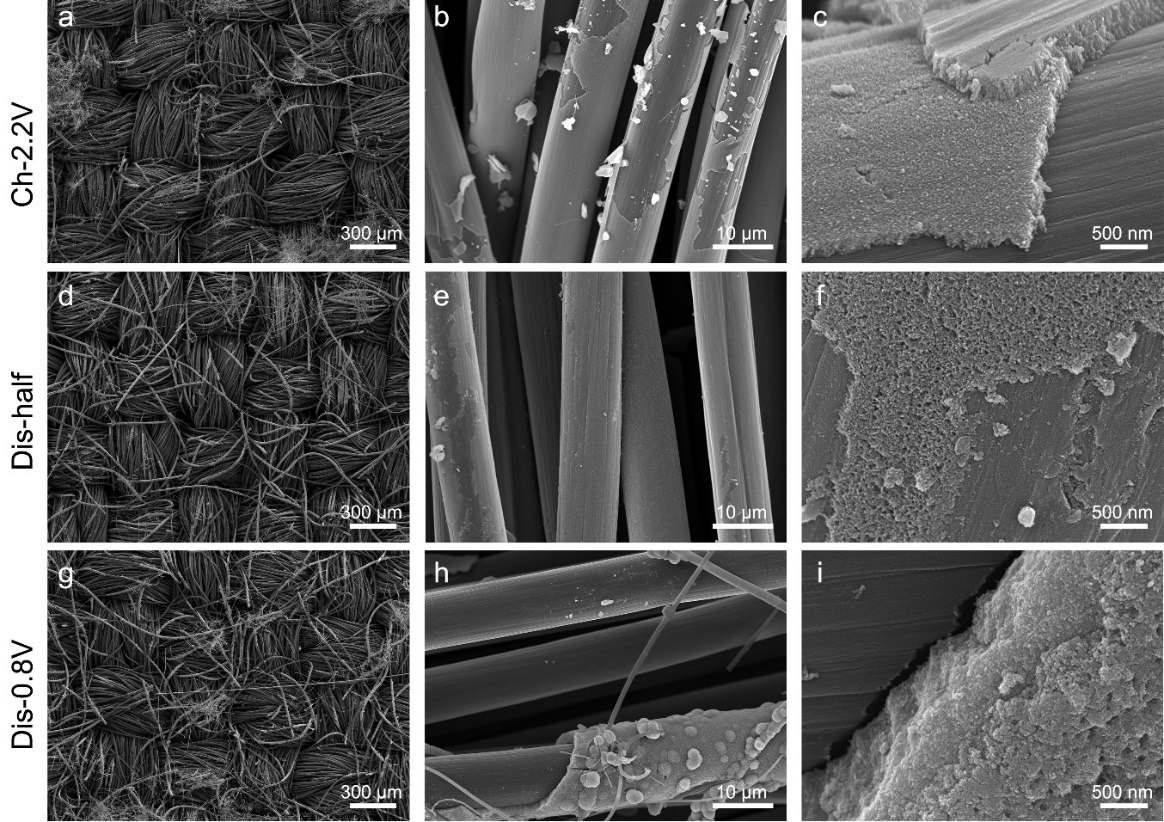


**Fig. S32** SEM images of the CE-MnO_2_ cell with AEM after 20 cycles at different states. (**a-c**) Ch-2.2V, (**d-f**) Dis-half and (**g-i**) Dis-0.8V


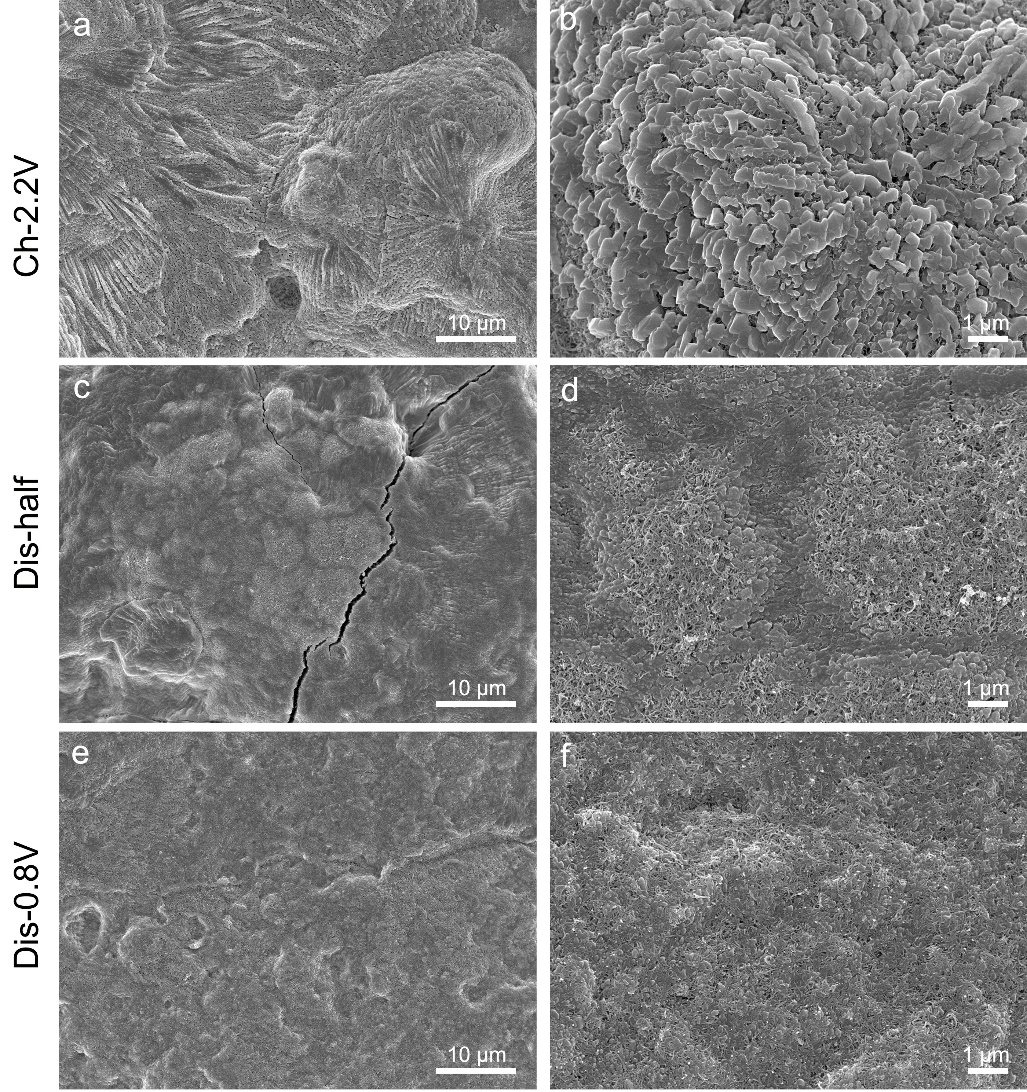


**Fig. S33** SEM images of the MnO_2_ slurry cell after 20 cycles at different states. (**a, b**) Ch-2.2V, (**c, d**) Dis-half and (**e, f**) Dis-0.8V


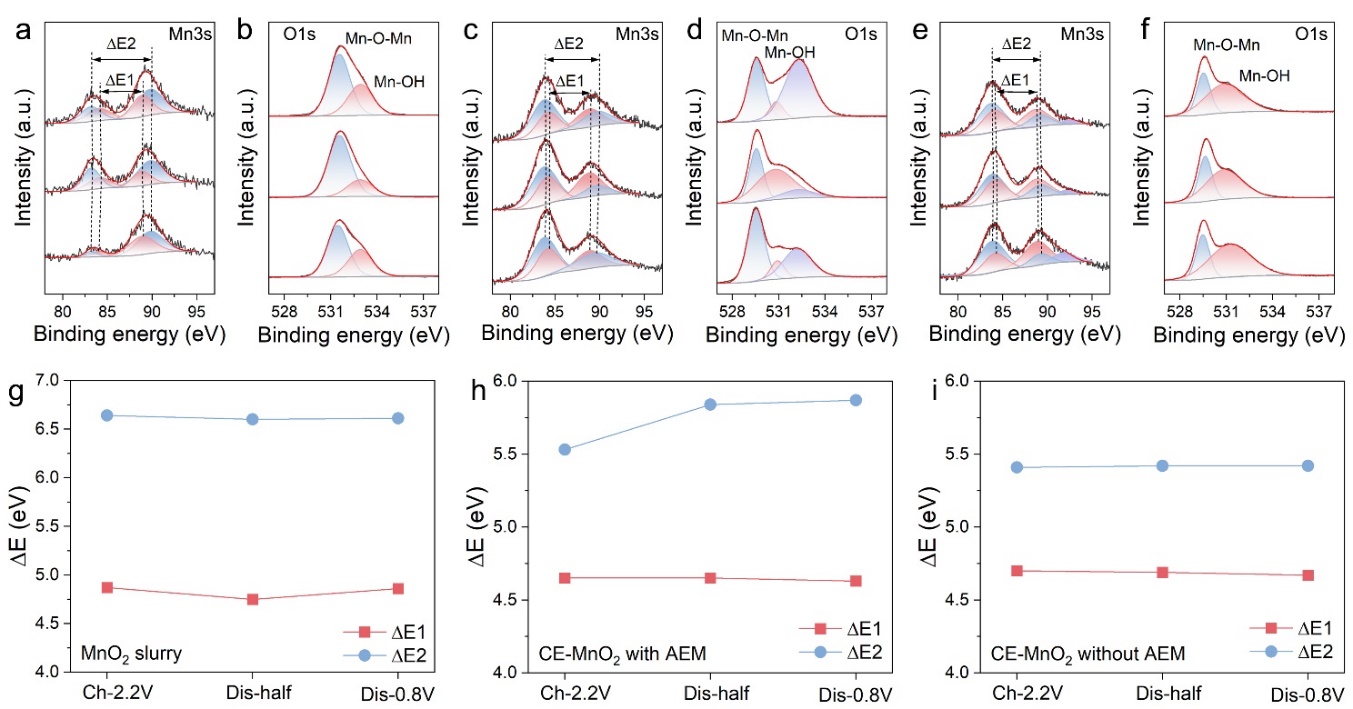


**Fig. S34** Mn 3s and O 1s spectra of (**a, b**) MnO_2_ slurry and CE-MnO_2_ cell (**c, d**) with and (**e, f**) without AEM after 20 cycles at different states. ∆E value of (**g**) MnO_2_ slurry and CE-MnO_2_ cells (**h**) with and (**i**) without AEM after 20 cycles at different states


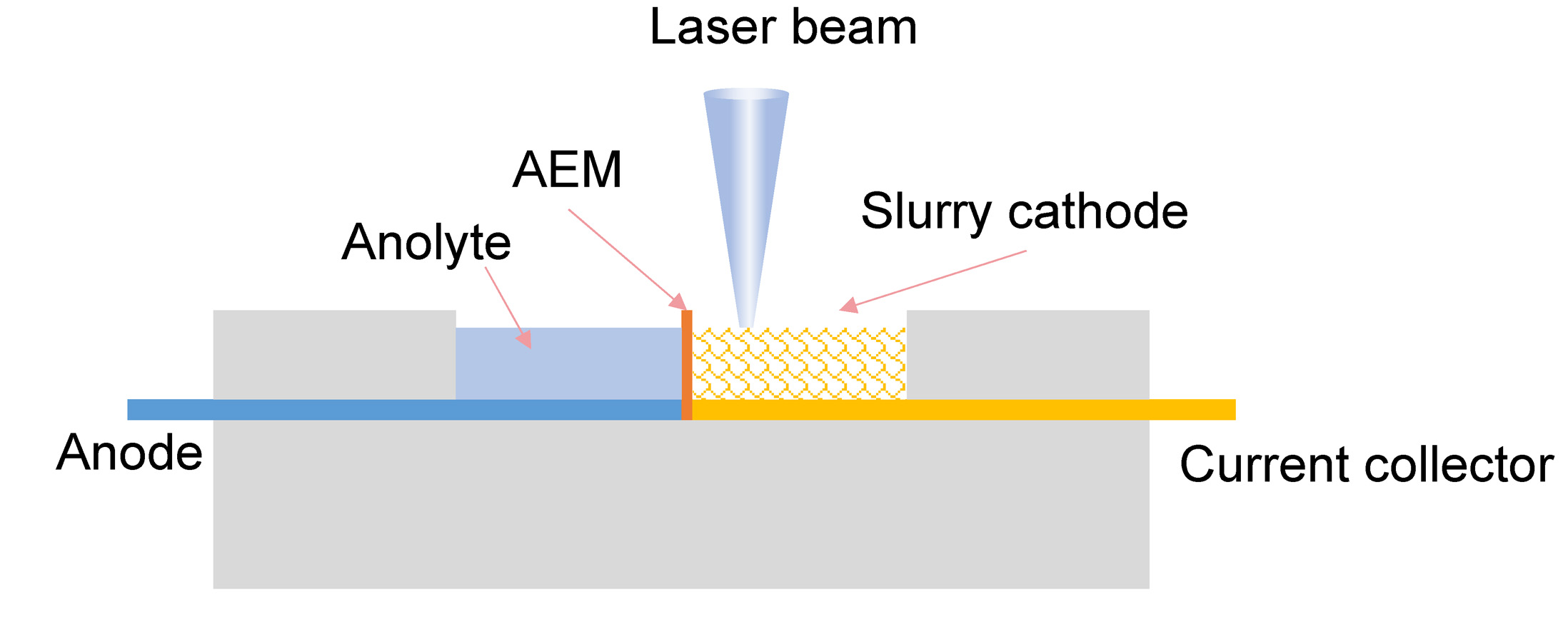


**Fig. S35** The schematic diagram of the electrolytic Zn-MnO_2_ slurry reaction tank for in-situ Raman spectroscopy record


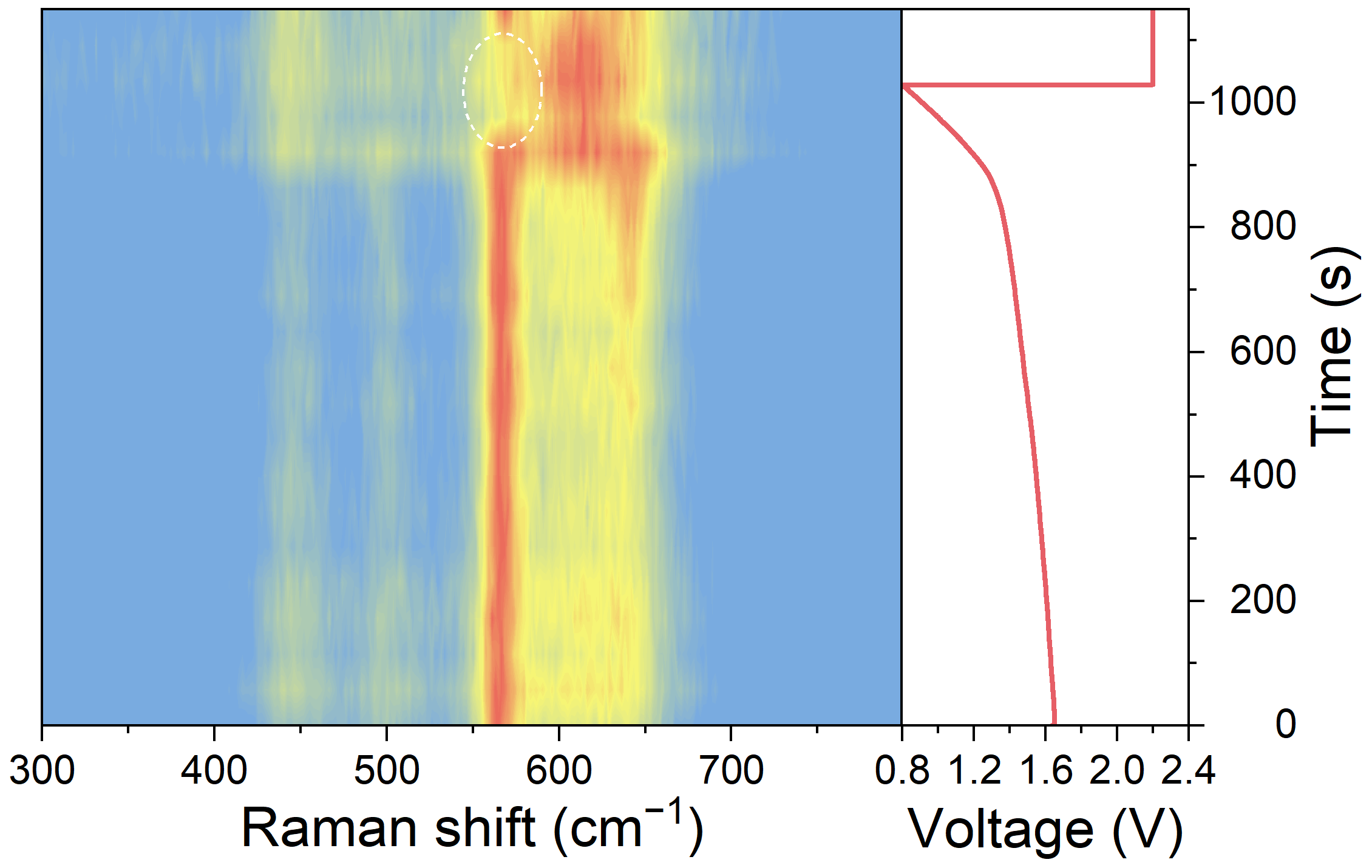


**Fig. S36** Contour map of in-situ Raman spectra and voltage curves of CE-MnO_2_ cells with AEM during discharge and charge


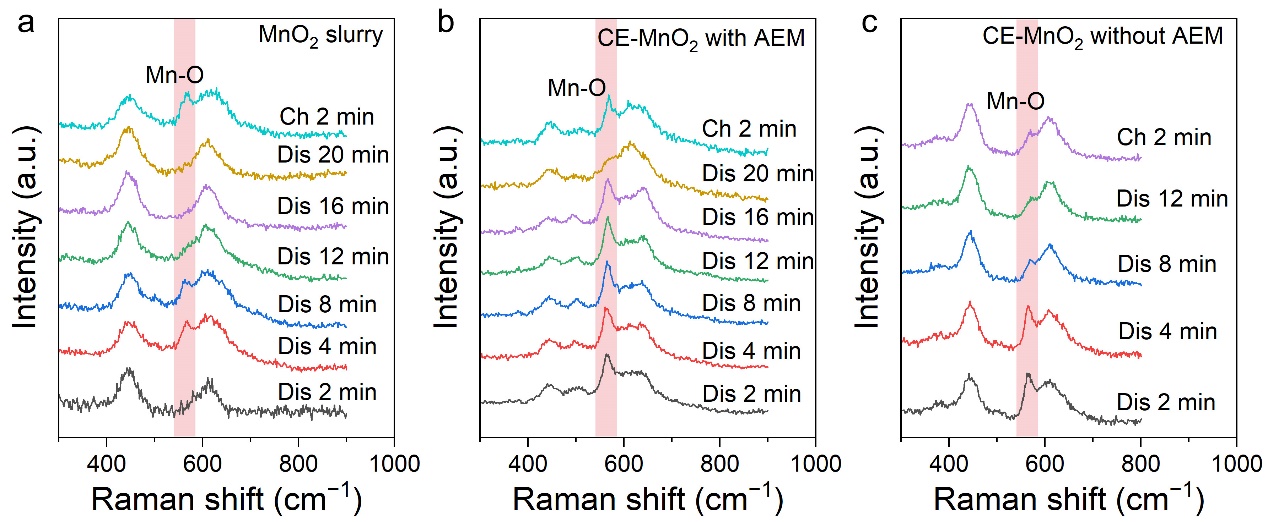


**Fig. S37** In-situ Raman spectra of (**a**) MnO_2_ slurry and CE-MnO_2_ cells (**b**) with and (**c**) without AEM at different charge and discharge states


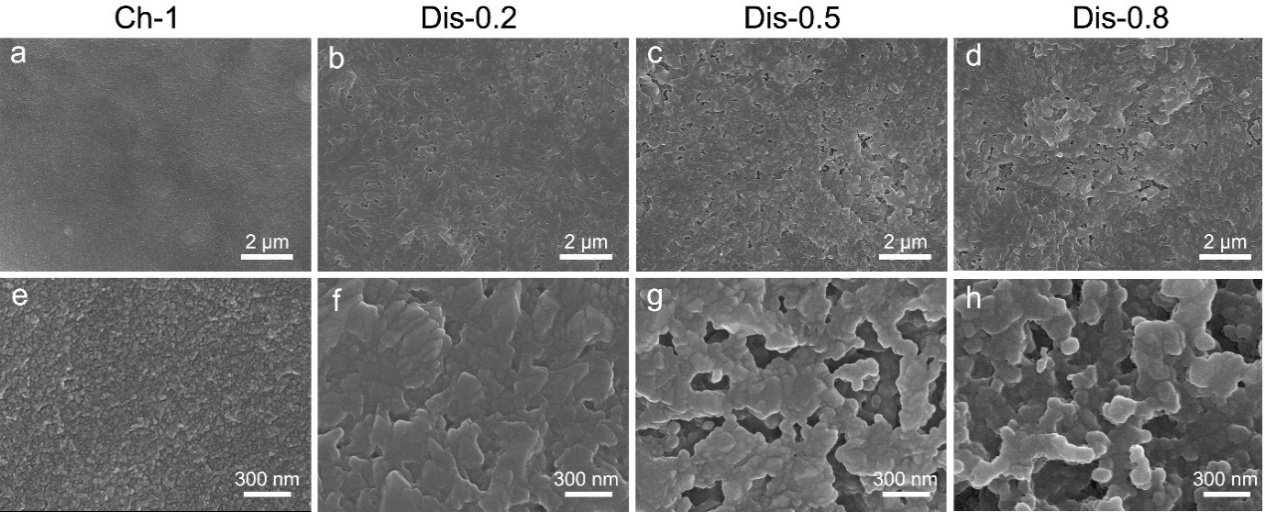


**Fig. S****38** SEM images of MnO_2_ deposition/dissolution on carbon paper at different states. (**a, e**) Ch-1 represents a charge for a capacity of 1 mAh cm^−2^; (**b, f**) Dis-0.2, (**c, g**) Dis-0.5 and (**d, h**) Dis-0.8 represent discharge for the capacities of 0.2 and 0.5 mAh cm^−2^ and to 0.8 V, respectively


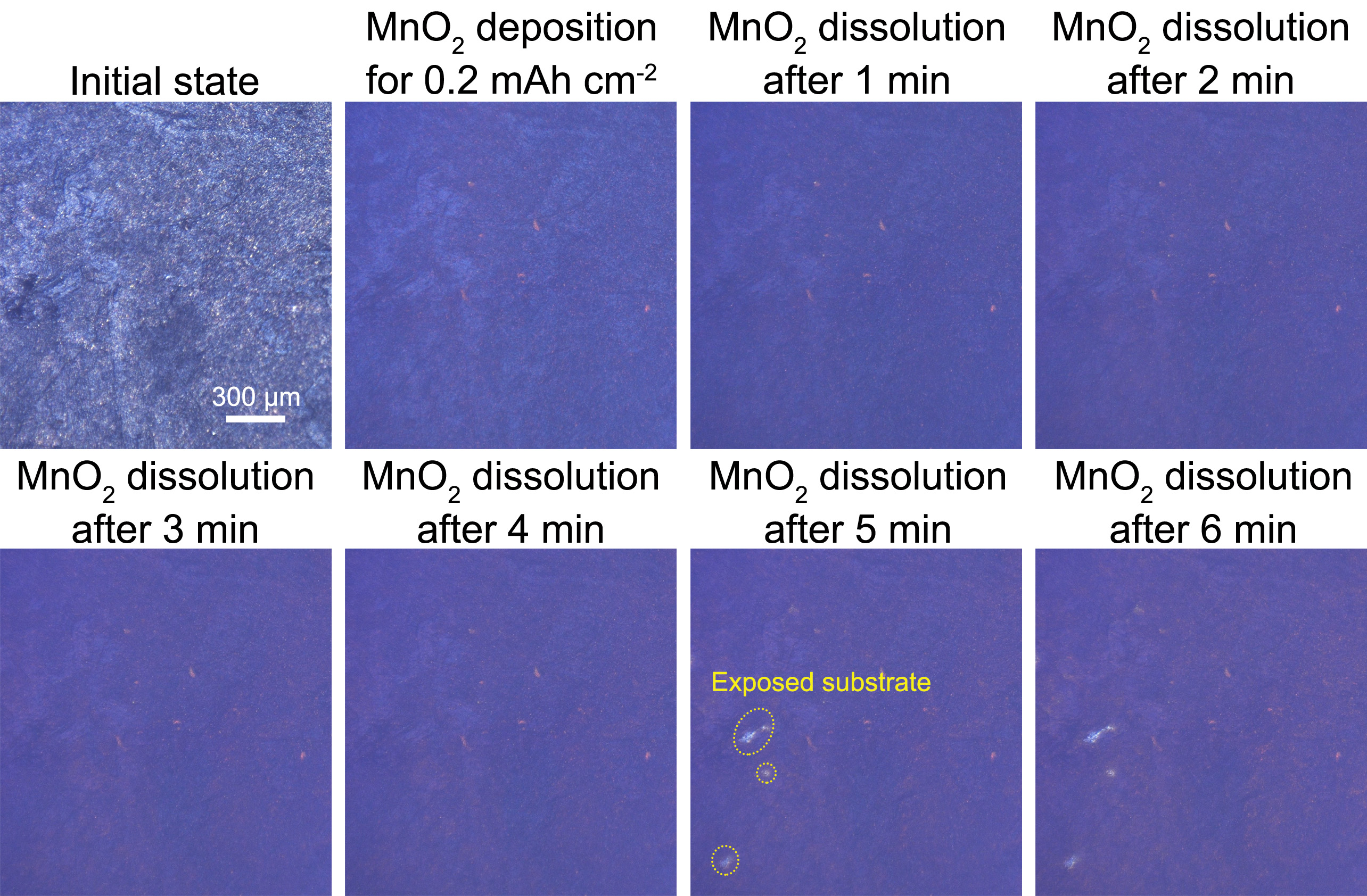


**Fig. S39** In-situ optical observations of MnO_2_ deposition/dissolution on carbon paper at different states


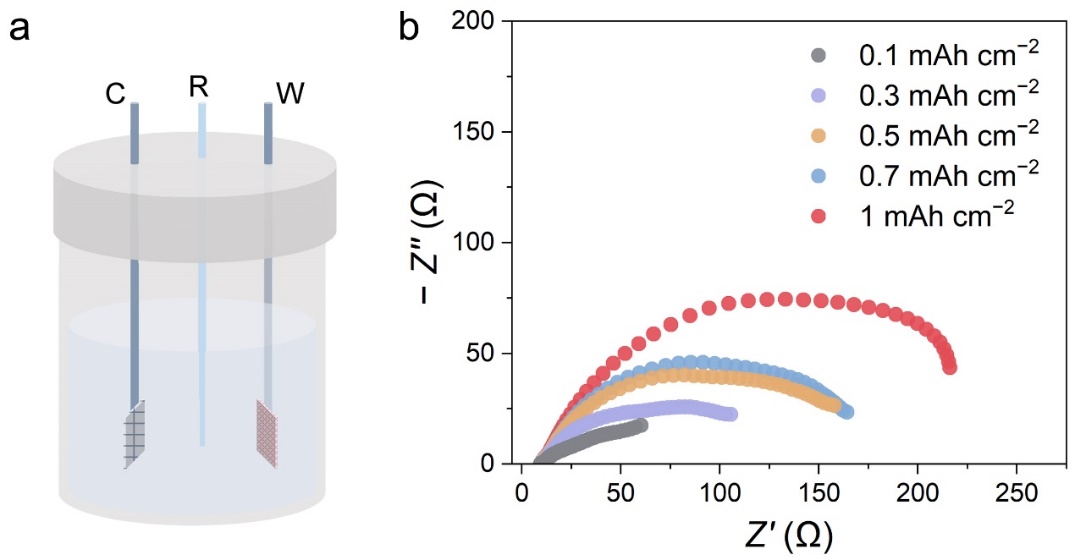


**Fig. S40** (**a**) Three electrode configurations with carbon cloth as working electrode, platinum mesh as a counter electrode and Ag/AgCl as a reference electrode for in-situ EIS measurements of MnO_2_ deposition for different capacities. (**b**) In-situ Nyquist plots of MnO_2_ deposition in three electrodes at different capacities


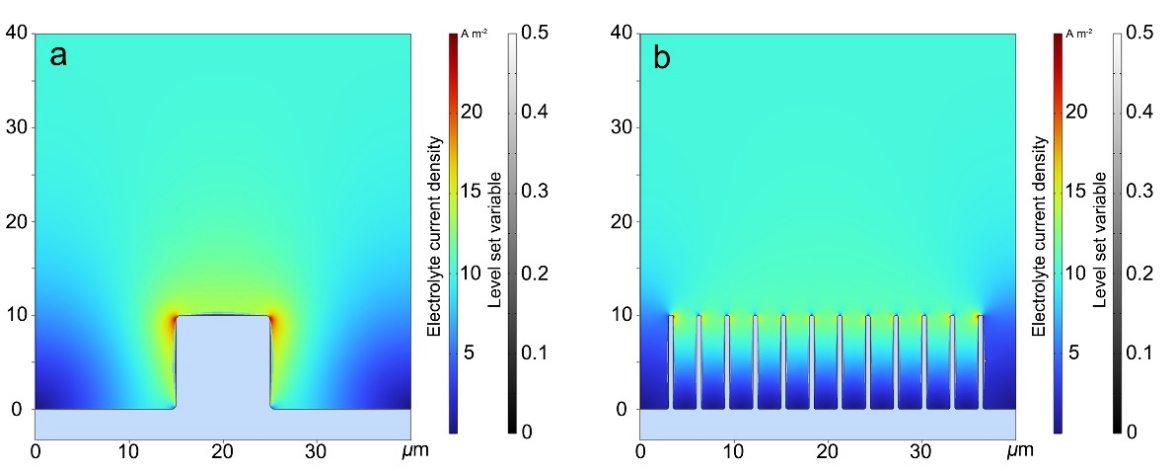


**Fig. S41** The electric field simulation of MnO_2_ deposition on (**a**) carbon cloth and (**b**) CNTs at initial state


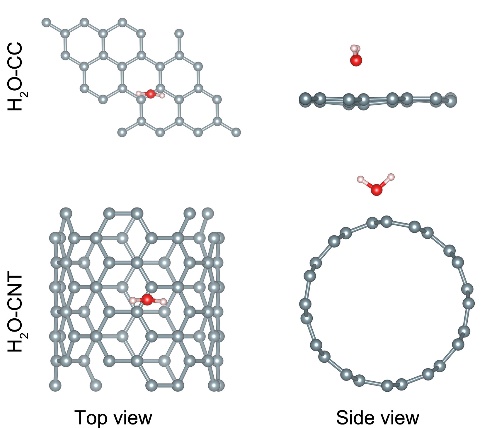


**Fig. S42** The structural models water molecules adsorbed on CC and CNTs


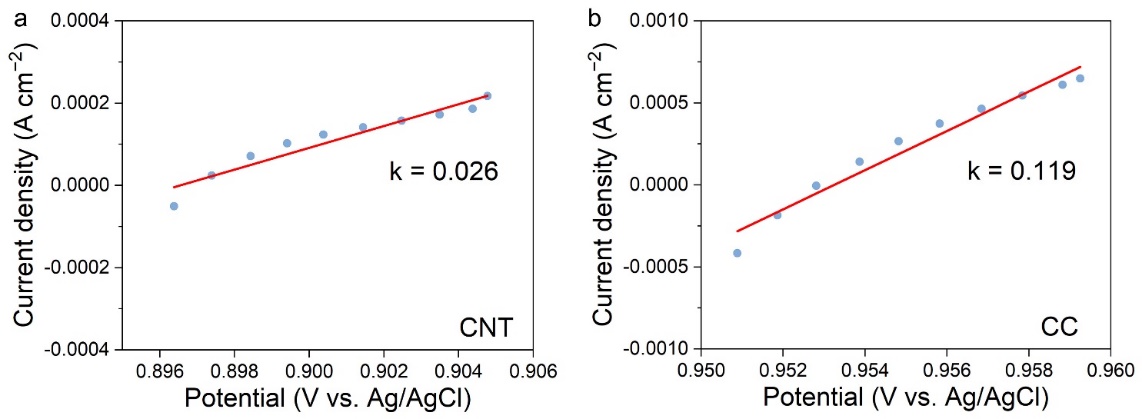


**Fig. S43** Linear polarization curves of (**a**) CNTs and (**b**) CC electrodes

The exchange current density of CNTs and CC electrodes was determined from the slope of the linear polarization curves according to follow Equation [S3]

$I_{0}=\frac{I_{d}RT}{F\eta}$ (S3)

where *I*_d_, *F*, *R*, *T* and *η* represent the applied current density, the Faraday constant (96500 C mol^−1^), the gas constant (8.314 J mol^–1^ K^–1^), the temperature (298 K), and the total overpotential (V), respectively.


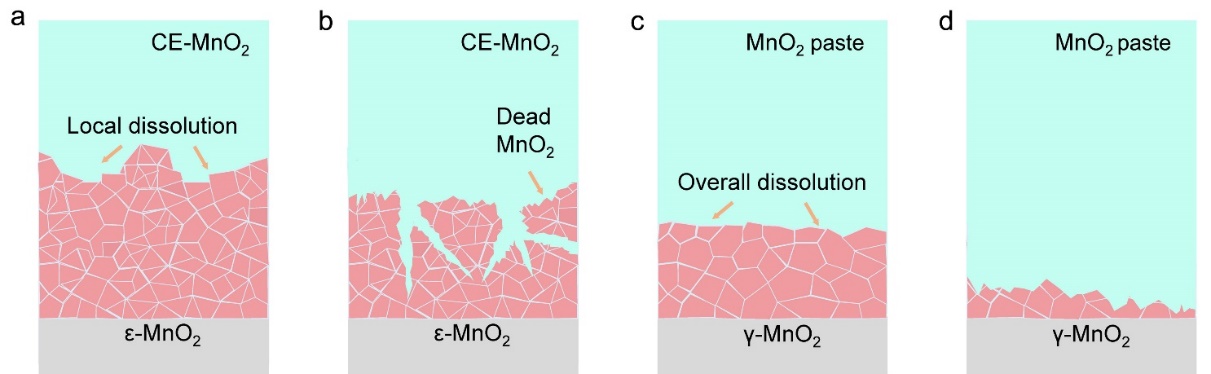


**Fig. S44** Schematic illustration of localized dissolution in the (**a, b**) CE-MnO_2_ cells and **(c, d**) overall dissolution in the MnO_2_ slurry cells

**Table S1** Parameters of MnO_2_ deposition and dissolution simulation in COMSOL

| Parameter | Symbol | Value | Unit |
| --- | --- | --- | --- |
| Initial Mn concentration | C_Mn2+,ref_ | 2000 | mol m^−3^ |
| Temperature | *T*_0_ | 298 | K |
| Exchange current density | *i*_0_ | 0.49 | A m^−2^ |
| Equilibrium potential | *E*eq | 0 | V |
| Anode potential | *φ*_s_anode_ | −1.23 | V |
| Cathode potential | *φ*_s_cathode_ | 1.23 | V |
| Cathode transfer coefficients | *α*_c_ | 1.5 |  |
| Anode transfer coefficients | *α*_a_ | 0.5 |  |
| Density of MnO_2_ | *ρ* | 0.08693 | kg mol^−1^ |
| Molar mass of MnO_2_ | *M* | 5.03 | g cm^3^ |
| Applied current density | appl_*i* | 1 | mA cm^−2^ |
| Mn^2+^ charge | *z*_Mn_ | 2 |  |

**Supplementary References**

1. C.M. Feng, D.D. Liu, Y.C. Chen, J.J. Du, P. Zhang, Evaluation method of the conductive property of the electrode slurry in semi-solid lithium battery. J. Funct. Mater. **48**(5), 5011–5016 (2017). <https://doi.org/10.3969/j.issn.1001-9731.2017.05.003>
2. C. Feng, Y. Chen, D. Liu, P. Zhang, Conductivity and electrochemical performance of LiFePO_4_ slurry in the lithium slurry battery. IOP Conf. Ser. Mater. Sci. Eng. **207**(1), 012076 (2017). <https://doi.org/10.1088/1757-899X/207/1/012076>
3. P.H.L. Notten, P. Hokkeling, Double-phase hydride forming compounds: a new class of highly electrocatalytic materials. J. Electrochem. Soc. **138**(7), 1877–1885 (1991). <https://doi.org/10.1149/1.2085893>
